# Compositional analysis of coffee containing javamide I/II and investigation of health effects in rats fed a high fat diet

**DOI:** 10.1038/s41598-025-13590-3

**Published:** 2025-08-06

**Authors:** Jae B. Park, Renee Peters

**Affiliations:** USDA, ARS, BHNRC, Diet, Genomics, and Immunology Laboratory, Bldg. 307C, Rm. 227, Beltsville, MD 20705 USA

## Abstract

Coffee containing javamide I/II (CCJ12) is commonly found in the market. However, no information is available about chemical composition of CCJ12 and in vivo effects on obesity. Therefore, in this paper, the composition of CCJ12 was analyzed by HPLC and LC/MS, and effects on bodyweight, metabolic (HDL, LDL, TG, leptin, adiponectin), cardiovascular risk (sE-selectin, C-reactive protein,), and inflammatory (MCP-1, TNF-alpha) factors were investigated in a rodent model. In CCJ12, > 700 compounds were identified by LC/MS and the amounts of javamide I/II, caffeine and chlorogenic acids were quantified by HPLC. For the animal study, rats were placed into three groups (each n = 10); (CG group (a control diet with water), FG group (a high fat diet with water), and FCG group (a high fat diet with CCJ12)) and the study was conducted for 20 weeks. The data showed no significant differences in water/food intakes between all three groups. However, the FG and FCG groups showed weight gain, in comparison to the CG group (*P* < 0.05). Also, the FG and FCG groups showed higher levels of LDL, TG, and leptin than the CG group (*P* < 0.05). However, no significant differences were found in bodyweight, HDL, LDL, TG, leptin, and adiponectin levels between the FG and FCG groups. Also, no significant differences were noted in sE-selectin, C-reactive protein, and MCP-1 levels between the FG and FCG groups. However, TNF-alpha level was found to be down in the FCG group, in comparison to the FG group (*P* < 0.05). Our study suggests that CCJ12 may have no adverse effects on bodyweight, HDL, LDL, TG, adiponectin, leptin, sE-selectin, C-reactive protein, and MCP-1, but may have a beneficial effect on TNF-alpha in rats fed a high fat diet.

**Supplementary Information:**

The online version contains supplementary material available at 10.1038/s41598-025-13590-3.

## Introduction

Worldwide, more than 600 million people are considered obese, and the obesity rate is likely to increase more in the future^1–6^. Furthermore, increased obesity may have adverse impacts on many diseases such as cardiovascular disease, diabetes, kidney disease, hypertension, fatty liver disease, osteoarthritis, and cancers^7–14^. Although lifestyles, high-calorie diets, and genetic traits are regarded as major contributing factors for increasing obesity, some compounds in foods and drinks are also alleged to contribute to increasing obesity^13–15^. Coffee is one of the popular drinks consumed worldwide. Many reports indicate that coffee consumption may have some positive effects on several human diseases including cognitive functions and diabetes^16–20^. Although coffee is largely considered beneficial to human health, there are also reports pointing out that further studies should be carried out to assess potential health effects of different types of coffee products and their compounds^17–21^. In fact, coffee beans (Arabica and Robusta beans) contain a great number of phytochemicals, and their contents are likely changed by many factors. Among them, weather change is considered as a significant factor impacting not only the cultivation but also the chemical contents of coffee beans^22,23^.

Consequently, it is likely that coffee beans and products with different amounts of coffee chemicals are available on the market. As expected, our previous studies reported that coffee products found in the market may contain different amounts of coffee chemicals (e.g., caffeine, chlorogenic acids, javamide I/II)^24–27^. Among the chemicals, javamide I/II (*N*-coumaroyltryptophan and *N*-caffeoyltryptophan) are phenolic-conjugated tryptophan compounds found in coffee beans^24,25^. Even though javamide I/II are present in both Arabica and Robusta beans, the data suggests that the amounts of javamide I/II can be higher in Robusta beans than Arabica beans^24–26^. Also, several studies indicated that javamide I/II and derivatives may have several important biological activities related to human health^27–35^. Particularly, they were reported to inhibit inflammatory cytokines better than caffeine^35^. In addition, a variety of coffee products containing javamide I/II (CCJ12) are commonly found in the market^24–27,34,35^. However, no information was available about potential effects of CCJ12 on obesity, although CCJ12 was previously studied in a non-obesity model^34^. Furthermore, the chemical composition of CCJ12 has not been investigated entirely. Therefore, in this study, CCJ12 was first analyzed by LC/MS and HPLC methods. Then, in vivo effects of CCJ12 on several obesity-related factors such as bodyweight, metabolic, cardiovascular, and inflammatory factors were investigated in rats fed a high fat diet. Potential effects of CCJ12 on bodyweight and metabolic factors (e.g., total cholesterol, HDL, LDL, triglycerides (TG)) were first investigated in this study because these factors are often considered as primary health concerns for many people including coffee drinkers^19–21^. Then, potential effects of CCJ12 on adipokines (leptin and adiponectin), cardiovascular (sE-selectin and C-reactive protein), and inflammatory (MCP-1 and TNF-alpha) factors were studied, because these factors are also associated with obesity as well as other diseases^36–51^. Especially, daily intakes of javamide I/II were calculated with those of caffeine and chlorogenic acids to evaluate their contributing effects on the biomarkers examined in this study, because many previous coffee studies lack this information, making it difficult to determine overall contributing effects^17–21^. Overall, in this study, important information was provided about HPLC/LC–MS data of CCJ12 and in vivo effects on bodyweight, HDL, LDL, TG, adiponectin, leptin, sE-selectin, C-reactive protein, MCP-1 and TNF-alpha in rats fed a high-fat diet.

## Materials and methods

### Materials

Caffeine, chlorogenic acids (5-caffeoylquinic acid (5-CQA), 4-caffeoylquinic acid (4-CQA), and 3-caffeoylquinic acid (3-CQA)), and other chemicals were obtained from Sigma Chemical Co. (St. Louis, MO), and javamide I/II standards were prepared as stated previously^24,25^. Coffee products were procured from internet suppliers. For the animal study, a control diet (AIN-76A purified diet) and a high fat diet (45% kcal from fat diet) were acquired from TestDiet (St. Louis, MO, USA).

### HPLC analysis

Caffeine, javamide I/II, 5-CQA, 4-CQA, and 3-CQA in CCJ12 were quantified using a high-performance liquid chromatography (HPLC) method^24,25^. Briefly, Nova-Pak C18 (Waters, Milford, MA, USA; 150 mm × 2.1 mm i.d., 4 µm) and a gradient condition were utilized; for 0–5 min, buffer A (20 mM NaH_2_PO_4_, pH 4.3), for 5–18 min, a first gradient from buffer A to buffer B (40% acetonitrile), for 18–25 min, a second gradient from buffer B to buffer C (60% acetonitrile) and for 5 min, buffer C at the flow rate (1 mL/min). Caffeine, javamide I/II, 5-CQA, 4-CQA, and 3-CQA were prepared in buffer A and the samples (10µL) were injected using an autosampler into the Agilent 1260 LC system (Agilent technologies, Santa Clara, CA, USA). The peaks of javamide I/II, caffeine, 5-CQA, 4-CQA, and 3-CQA were detected using a photo diode array detector (DAD) (Agilent technologies, Santa Clara, CA, USA).

### LC/MS analysis

CIL-LC/MS (chemical isotope-labelling-LC/MS) (Agilent 1290 LC linked to Bruker Impact II Q-tof mass spectrometer) was run as stated previously^35^. Briefly, an Agilent eclipse plus reversed-phase C18 column (150 × 2.1 mm, 1.8 µm particle size) was employed with mobile phases A (0.1% (v/v) formic acid in water) and B (0.1% (v/v) formic acid in acetonitrile) following a gradient of t = 0 min, 25% B; t = 10 min, 99% B; t = 13 min, 99% B; t = 13.1 min, 25% B; and t = 16 min, 25% B at a flow rate of 400 µL/min and a column chamber temperature of 40 ℃. To monitor the instrument performance, QC samples were tested every 9 sample runs, and calibration data were utilized to monitor the retention time. To find out coffee chemicals in CCJ12, LC–MS data from 4-channels was converted into .csv file with Bruker Data Analysis 4.4. Then, the exported data was analyzed using IsoMS Pro 1.2.3 software. Ions/peak pairs absent in < 80% of samples were deleted from the data to assure data quality, and the data were normalized using the ratio of total useful signals. Coffee chemicals were identified using a two-tier ID approach. In tier 1, peak pairs were matched against an in-house standards metabolite library based on triple parameters (accurate molecular masses, retention times, and MSM/MS spectra). In tier 2, the remaining peak pairs were identified using a linked identity library which includes > 7000 pathway related metabolites.

### Preparation of CCJ12

For the study, CCJ12 was prepared using Robusta-blend coffees as reported previously^34^. Briefly, CCJ12 (30 ml) was prepared to contain javamide I (0.048 mg), javamide II (0.36 mg), caffeine (12 mg) and chlorogenic acids (10 mg). The doses of javamide I/ II were calculated by the dose conversion factor which is suggested in the FDA guideline “Estimating the Maximum Safe Starting Dose in Initial Clinical Trials for Therapeutics in Adult Healthy Volunteers” (https://www.fda.gov/regulatory-information/search-fda-guidance-documents/estimating-maximum-safe-starting-dose-initial-clinical-trials-therapeutics-adult-healthy-volunteers), as reported previously^34^.

### Animal study

Sprague–Dawley male rats (8-week-old) (n = 30) were acquired from Charles River (Wilmington, MA, USA). Rats were placed in ventilated micro-isolator racks with an automatic watering system located in a room with a 12:12-h light/dark cycle and ambient temperature of 23–18 °C with relative humidity of 55.5% for two-week acclimatization. After that, the animal study was carried out according to the animal protocol approved by the Beltsville Area Animal Care and Use Committee (AUP Approval No. 16–020) which was in accordance with the Institutional Animal Care and Use Committee (IACUC). Also, this study was performed in accordance with relevant guidelines and regulations, and all methods are reported in accordance with ARRIVE guidelines. At the time of the study, rats were 10-week-old and had bodyweights of 175.9 ± 19.3 g (mean ± SEM). Rats were placed into three groups: CG group (a control diet (AIN-76A purified diet) with drinking water (n = 10), FG group (a high fat diet (45% kcal from fat diet) with drinking water (n = 10), and FCG group (a high fat diet with drinking water containing CCJ12 (n = 10)). The number of animals (n = 10) was set based on the previous data that each group (n = 10) provided a power of > 0.99 able to detect a 20% difference in means of outcome variables with a standard deviation of 15%^34^. The study was conducted for 20 weeks. During the study, CCJ12 was supplied to the FCG group, meanwhile water was supplied to the CG and FG groups. Food/water intakes and bodyweight were monitored weekly. On the last day, after 18 h fasting, rats were individually euthanized by CO_2_ gas. Upon the absence of vital signs (e.g., heartbeat, respiration, and response to stimuli), each rat was removed from the cage and blood was drawn, transferred into EDTA-coated vials and centrifuged (3300 rpm for 15 min) for plasma samples which were stored at − 80 °C.

### LDL, HDL, cholesterol, and total triglyceride assays

Triglyceride (TG; Cat. #10010303), total cholesterol (TC; Cat. #10007640), and high-density lipoprotein cholesterol (HDL: Cat. # ab65390) were determined in plasma samples using respective assay kits (Cayman Chemical Inc, Ann Arbor, MI and Abcam, Cambridge, MA). Low-density lipoprotein cholesterol (LDL) was estimated from the concentrations of TG, TC, and HDL using the modified Friedwald formula: LDL = TC-HDL-0.16*(TG).

### Leptin and adiponectin assays

Rat leptin ELISA kit (Cat. # EZRL-83 K; Millipore Corp., Billerica, MA) was used to determine the level of leptin in the plasma samples, according to the manufacturers’ protocols. Briefly, Assay was performed using 10ul plasma sample with background and standard controls. All procedures were performed at room temperature. After all procedures were done, the absorbance of samples was recorded at 450 nm and 590 nm using a plate reader, and the difference of absorbance was calculated to determine the level of plasma leptin. Rat total adiponectin ELISA kit (Cat. # RRP300; R&D Systems, Minneapolis, MN) was used to determine the level of adiponectin in plasma samples, according to the manufacturers’ protocols. Briefly, assay was performed using 50 µl plasma sample with background and standard controls at room temperature. After all procedures were completed, the absorbance of samples was recorded at 450 nm and 570 nm, the difference of absorbance was calculated, and the difference was used to determine the level of adiponectin.

### C-Reactive protein and sE-selectin assays

C-Reactive protein (CRP) in plasma samples was determined with rat CRP ELISA Kit (Cat. # ab108827; Abcam, Cambridge, MA). Briefly, assay was performed using 50 µl of plasma sample with background and standard controls at room temperature. After all procedures were completed, the absorbance of samples recorded at a wavelength of 450 nm was used to determine the level of CRP. sE-selectin in the samples was measured using rat sE-Selectin ELISA Kit (Cat. # ab171334; Abcam, Cambridge, MA) according to the manufacturers’ protocols. Assay was performed using 100 µl of plasma sample with background and standard controls at room temperature. After all procedures were done, the absorbance of samples recorded at 450 nm was used to determine the level of sE-selectin.

### TNF-alpha and MCP-1 assays

TNF-alpha levels of plasma samples were measured using rat TNF-alpha ELISA kit (Cat. # KRC3011; Thermos Fisher, Camarillo, CA) according to the manufacturers’ protocols. Briefly, assay was performed using 100 µl of plasma sample with background and standard controls at room temperature. This kit detects both endogenous and recombinant rat TNF-alpha without low cross-reactivity against other related proteins. MCP-1 levels of the plasma samples were determined by rat MCP-1 ELISA kit (Cat. # MJE00; R&D systems, Minneapolis, MN) according to the manufacturers’ protocols.

### Statistical analysis

Sigma Plot 11.0 (Chicago, IL) was used to conduct statistical analyses. One-way ANOVA with Holm-Sidak method was used to calculate *P* value, and *P* < 0.05 was deemed statistically significant. The Holm-Sidak method was used in this study because it can be used for both pairwise comparisons and comparisons verse a control group, and because it is more powerful than the Turkey and Bonferroni methods and is often recommended as the primary procedure for most multiple comparison testing. Data points were denoted as the mean ± SD (n = 10).

## Results

### HPLC analysis

CCJ12 was prepared for this animal study, as stated previously^34^. Then, the amounts of caffeine, javamide I/II and chlorogenic acids in CCJ12 were quantified by HPLC as described in “Materials and Methods”. The data showed that caffeine (12 mg), javamide I (0.048 mg), javamide II (0.36 mg), and chlorogenic acids (10 mg) were in CCJ12 (30 ml).

### LC/MS analysis

Untargeted compound analysis was performed to identify a broad range of coffee compounds in CCJ12, and the data was processed as described in “Materials and Methods”. In CCJ12, > 700 compounds were identified (Table S1). As expected, CCJ12 was found to contain chlorogenic acids and isomers (e.g., chlorogenic acid, cryptochlorogenic acid, isomer 1 of cryptochlorogenic acid, 1-O-caffeoylquinic acid, p-coumaroyl quinic acid/3-O-p-coumaroylquinic acid, isomer 1 of p-coumaroyl quinic acid/3-O-p-coumaroylquinic acid, isomer 1 of 3-O-feruloylquinic acid, isomer 2 of 3-O-feruloylquinic acid, isomer 3 of 3-O-feruloylquinic acid, 3-O-feruloylquinic acid). CCJ12 was also found to contain several precursors of chlorogenic acids (e.g., isomer 2 of 5-O-caffeoylshikimic acid/3-O-caffeoylshikimic acid, isomer 3 of 5-O-caffeoylshikimic acid/3-O-caffeoylshikimic acid, isomer 1 of 5-O-caffeoylshikimic acid/3-O-caffeoylshikimic acid, 5-O-caffeoylshikimic acid/3-O-caffeoylshikimic acid, and 4-coumaroylshikimic acid) (Table 1). In addition, chlorogenic acid-derived lactones (e.g., 4-p-coumaroyl-1,5-quinolactone, isomer 2 of 4-caffeoyl-1,5-quinolactone, isomer 3 of 4-caffeoyl-1,5-quinolactone, 4-caffeoyl-1,5-quinolactone, isomer 1 of 4-caffeoyl-1,5-quinolactone, 3-feruloyl-1,5-quinolactone) were detected in CCJ12 (Table 1). Also, trigonelline, N-caffeoyltyrosine and javamide II (N-caffeoyltryptophan) were detected in CCJ12 (Table 1).

**Table 1:** The list of some coffee compounds from LC/MS data.

Mass (Da)	Compound	Relative pk intensity
137.0485	Trigonelline	0.8185
180.0648	Theophylline	0.90075
336.0848	5-O-Caffeoylshikimic acid	4.08375
336.0848	Isomer 3 of 5-O-Caffeoylshikimic acid	1.3475
336.085	Isomer 2 of 4-Caffeoyl-1,5-quinolactone	0.56925
336.0852	Isomer 2 of 5-O-Caffeoylshikimic acid	1.113
336.0858	4-Caffeoyl-1,5-quinolactone	0.25025
336.0858	Isomer 1 of 4-Caffeoyl-1,5-quinolactone	1.87325
336.0875	Isomer 1 of 5-O-Caffeoylshikimic acid	0.9075
338.0997	p-Coumaroyl quinic acid	1.093
343.1056	Caffeoyltyrosine	0.49475
350.1004	3-Feruloyl-1,5-quinolactone	1.698
350.1016	4-Feruloyl-1,5-quinolactone	1.0125
354.0937	Isomer 1 of Cryptochlorogenic acid	2.57675
354.0951	Cryptochlorogenic acid	0.89775
354.0951	Chlorogenic acid	3.1465
354.0956	Isochlorogenic acid	1.17925
354.0969	1-O-Caffeoylquinic acid	0.72225
366.1224	Javamide-II (N-Caffeoyltryptophan)	0.136
368.1104	5-O-Feruloylquinic acid	0.733
368.1105	3-O-Feruloylquinic acid	0.4995
368.1106	Isomer 1 of 3-O-Feruloylquinic acid	0.592

The analysis was conducted as described in "Materials and Methods". Compound names, exact mass, and relative peak intensity were presented. For other coffee compounds, see Table S1.

### Animal study

**Fig. 1:**
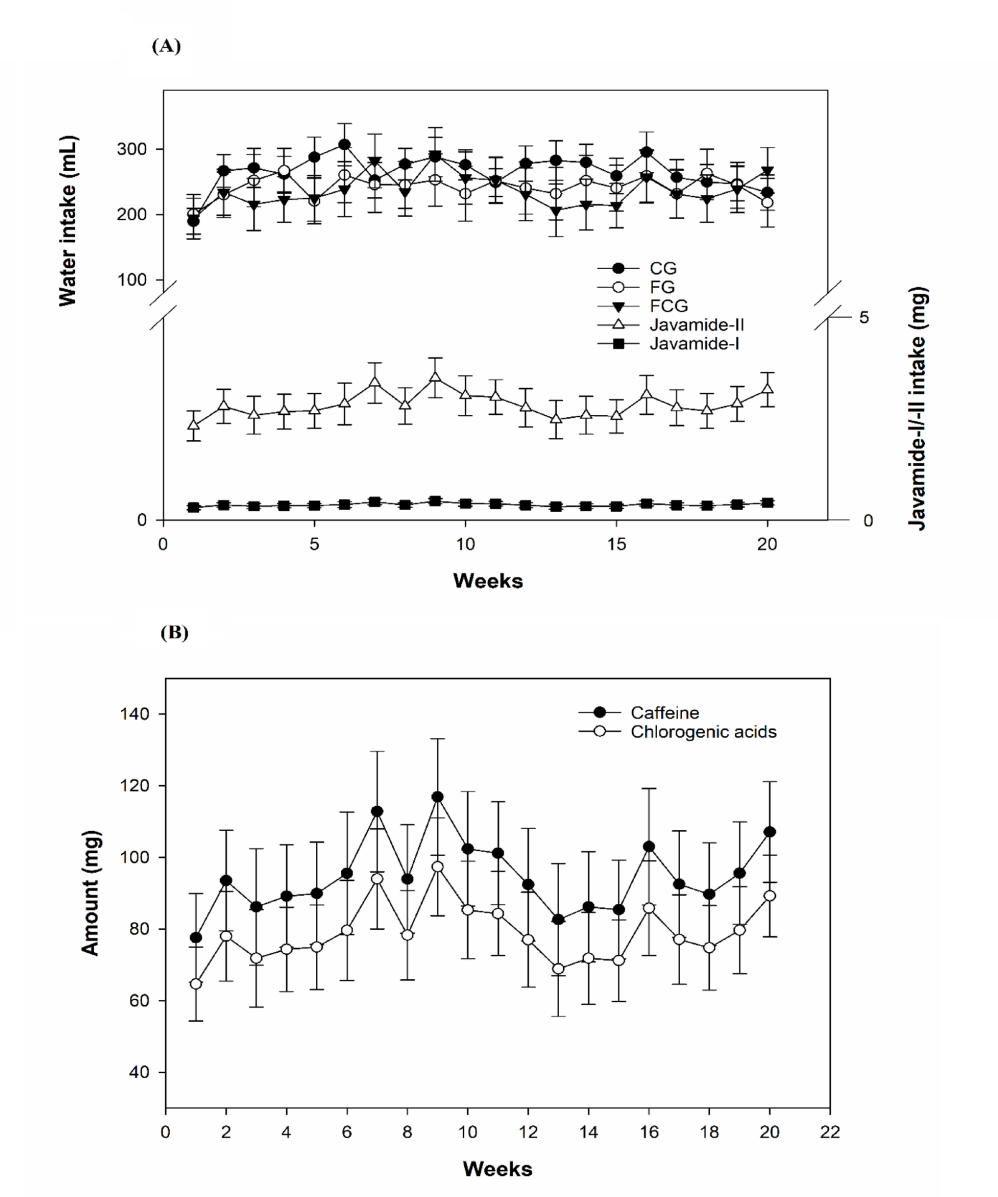
Water consumption and intakes of javamide I/II, chlorogenic acids and caffeine. CG, a control diet; FG, a high fat diet, FCG, a high fat diet with CCJ12. (**A**) The intakes of javamide I/II were calculated based on water consumption. (**B**) The intakes of chlorogenic acids and caffeine were calculated based on the water consumption. Data is detonated as the mean ± SD (n = 10).

**Fig. 2:**
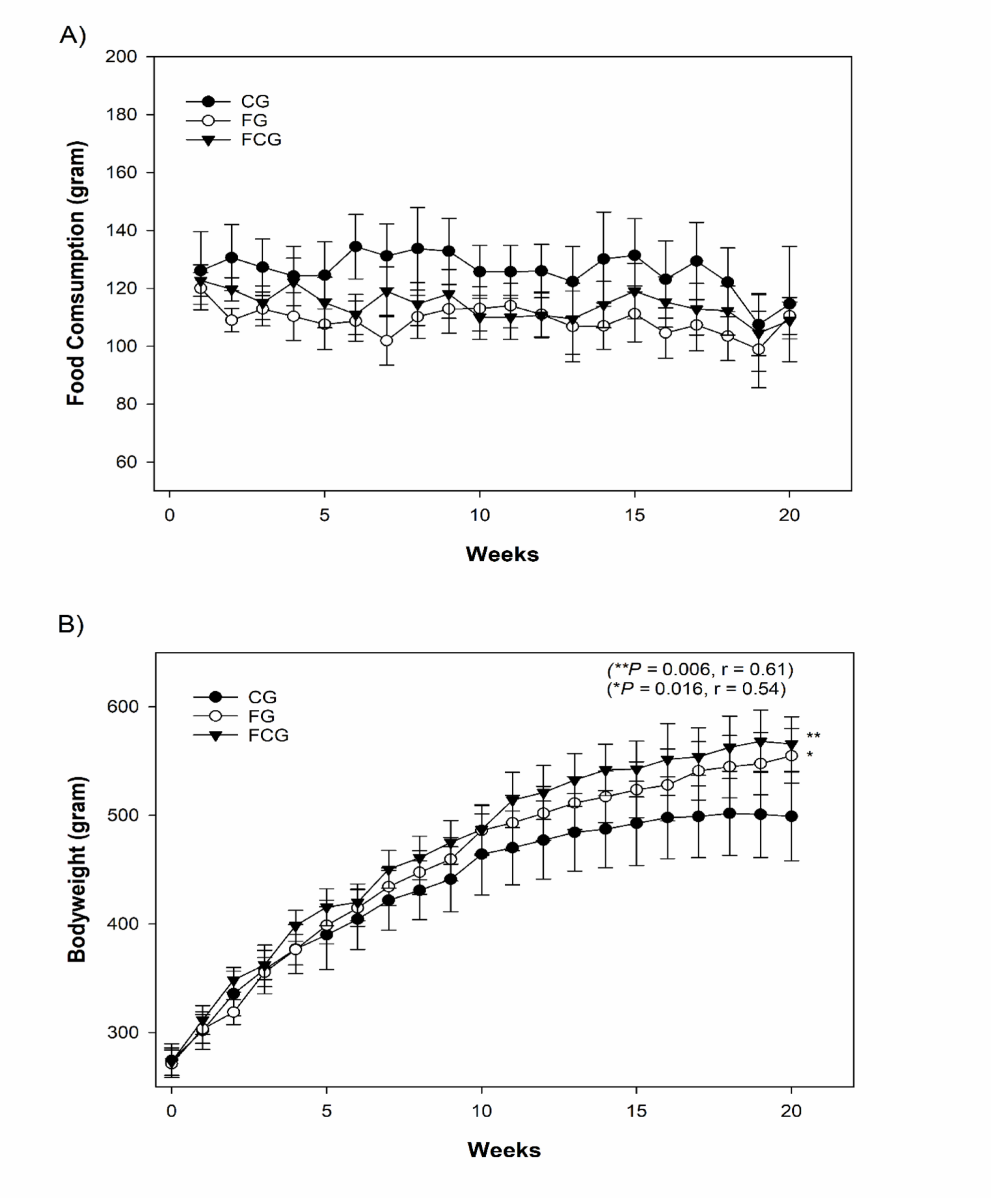
Chow consumption and bodyweight. (A) chow consumption and (B) bodyweight. CG, a control diet; FG, a high fat diet, FCG, a high fat diet with CCJ12. Data was analyzed using the one-way ANOVA with Holm-Sidak method. Data is detonated as the mean ± SD (n = 10), r is effect size, and the marks (* and **) indicate statistical significance compared to the CG group (*P* < 0.05).

Potential effects of CCJ12 on bodyweight and metabolic/cardiovascular/inflammatory factors were studied in a diet-induced obese rat model. In this study, rats were placed into three groups (CG, FG and FCG) as described in “Materials and Methods”. During the study, the intakes of water/food and bodyweight were monitored weekly. The weekly consumption of water/food was calculated by subtracting the remaining water/chow from the initial water/chow amounts (Fig. 1). Also, the bodyweight of each rat was individually measured weekly (Fig. 2). Additionally, illness/accidental death was monitored, and no illness/accidental death occurred during the study.

### Daily Intakes of javamide I/II, caffeine and chlorogenic acids

Using weekly water intake data, the daily intakes of javamide I/II, caffeine and chlorogenic acids were calculated (Fig. 1A). The data showed that the weekly average intakes of javamide I/II were around 0.37 and 2.84 mg, respectively (Fig. 1A). Based on this data, the daily intakes of javamide I/II were estimated to be around 0.05 and 0.40 mg. Because caffeine and chlorogenic acids were also in CCJ12, their daily intakes were also determined, and found to be about 13 and 11 mg, respectively (Fig. 1B). The data indicated that CCJ12 had little effect on water intake in the FCG group, in comparison to the CG and FG groups.

### Effects of CCJ12 on food intake and bodyweight

During the study, food intake was also monitored. All three groups showed a similar pattern of food intake during the study (Fig. 2A). Particularly, no significant difference was found in food intake between the FG and FCG groups, suggesting that CCJ12 may not induce or suppress the appetite for a high-fat diet in the FCG group, in comparison to the FG group. But there were significant weight gains in the FG and FCG groups, in comparison to the CG group, possibly due to a high fat diet (*P* < 0.05). However, the FG and FCG groups showed little variance in bodyweight (Fig. 2B), indicating that CCJ12 may not cause significant changes of food intake and bodyweight in the FCG group, compared to the FC group. The statistical analysis was performed using one-way ANOVA with the Holm-Sidak method. However, the outcomes were similar even if the analysis was conducted using other methods (Turkey or Bonferroni). Together, this data suggests that javamide I/II in CCJ12 may not cause significant changes of food intake and bodyweight in the FCG group, compared to the FG group.

### Effects of caffeine and chlorogenic acid on food intake and bodyweight

The data showed that the daily intakes of caffeine and chlorogenic acids were about 13 and 11 mg, equivalent to 293 and 248 mg in humans, respectively (Fig. 1B). Therefore, prospective effects of caffeine and chlorogenic acids in CCJ12 on food intake and bodyweight were also examined. No significant differences were found in food intake and bodyweight between the FG and FCG groups (Fig. 2), suggesting that caffeine and chlorogenic acids in CCJ12 may not induce significant changes of food intake and bodyweight in the FCG group, in comparison to the FG group.

### Effects of CCJ12 on HDL, LDL, and triglycerides

The levels of HDL, LDL, and triglycerides (TG) were determined in plasma samples from the CG, FG and FCG groups. The levels of HDL, LDL, and TG levels were significantly different in high fat-diet groups (the FG and FCG groups), in comparison to the CG group (Table 2). However, no significant difference was found between the FG and FCG groups, suggesting that CCJ12 may not induce significant changes of HDL, LDL and TG in rats fed a high-fat diet.

**Table 2:** Effects of CCJ12 on HDL, LDL and TG. TG (triglyceride), HDL (high-density lipoprotein cholesterol) and LDL (low-density lipoprotein). CG, a control diet; FG, a fat diet, FCG, a fat diet with CCJ12.

Lipids	CG	FG	FCG
LDL-C (mg/dL)	21 ± 1.9	27* ± 2.8	28* ± 3.5
HDL-C (mg/dL)	41 ± 4.1	35* ± 4.1	36* ± 4.9
TG (mg/dL)	102 ± 12.3	130* ± 15.2	132* ± 16.1

The data were analyzed using one-way ANOVA and were found statistically insignificant between the groups. Data are presented as the mean ± SD (n = 10) and the marks (*) indicate statistical significance compared to the CG group (*P* < 0.05).

### Effects of CCJ12 on adiponectin and leptin

**Fig. 3:**
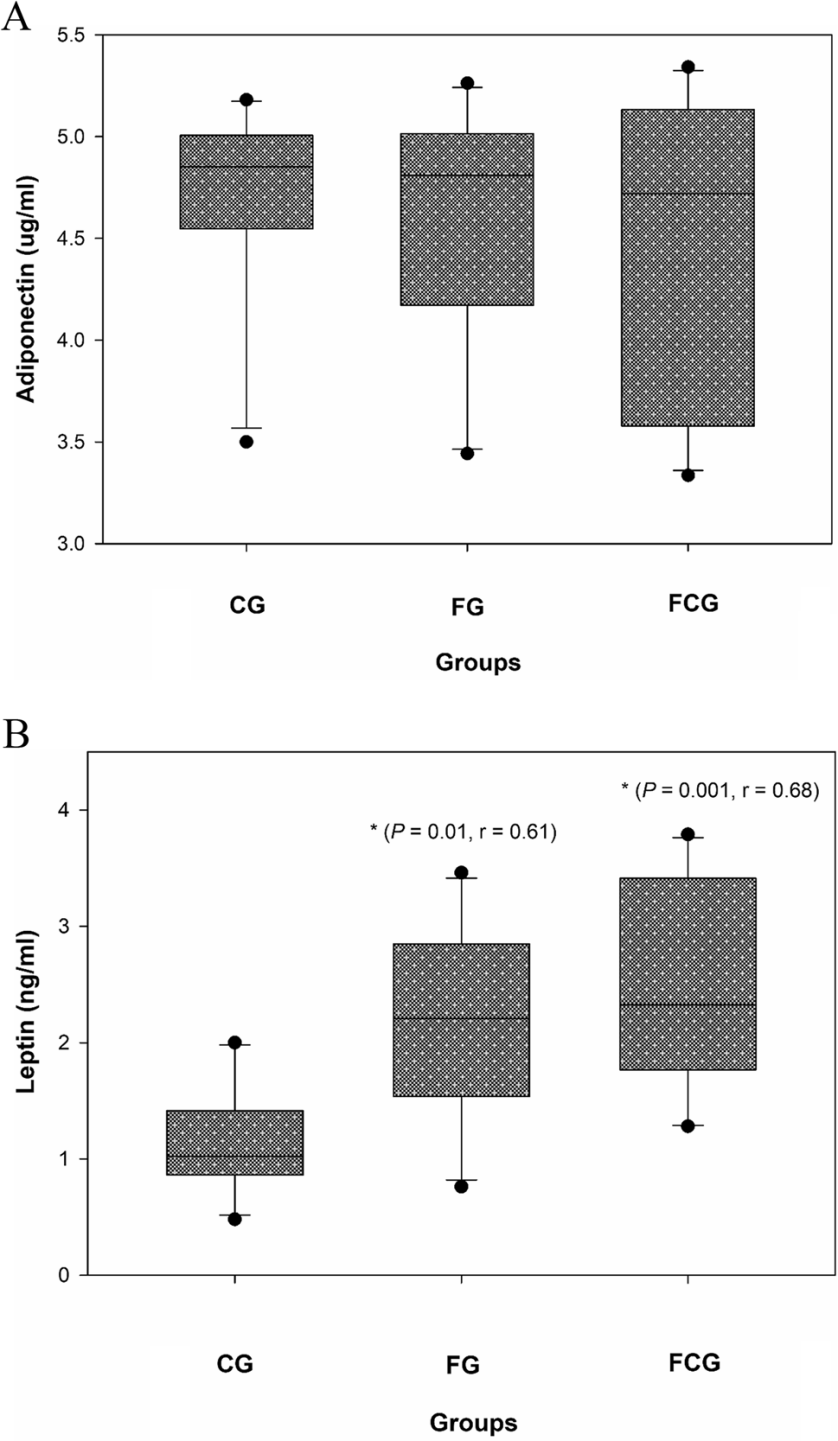
Effects of CCJ12 on adiponectin and leptin. Plasma adiponectin (**A**) and leptin (**B**) levels. CG, a control diet; FG, a high fat diet, FCG, a high fat diet with CCJ12. The data was analyzed using the one-way ANOVA with Holm-Sidak method. Data is detonated as the mean ± SD (n = 10), r is effect size, and the marks (*) indicate statistical significance compared to the CG group (*P* < 0.05).

Adiponectin and leptin levels were determined in the samples, because these adipokines are commonly used as reliable biomarkers for obesity^36–38^. As shown in Fig. 3A, the data showed a small reduction in adiponectin mean levels in the FG and FCG groups, in comparison to the CG group, even though the difference turned out to be insignificant. Also, no significant difference was found in adiponectin level between the FG and FCG groups, indicating that CCJ12 may not provide a substantial effect on adiponectin in the FCG group, in comparison to the FG group (The Turkey or Bonferroni methods also provided the similar outcomes). However, leptin levels were found to be higher in the FG and FCG groups, in comparison to the CG group (Fig. 3B). In fact, this data confirmed bodyweight gains in the FG and FCG groups, in comparison to the CG group (*P* < 0.05). However, no significant difference was found in leptin level between the FG and FCG groups, suggesting that CCJ12 may not produce a significant change of leptin level in the FCG group, in comparison to the FG group.

### Effects of CCJ12 on C-reactive protein (CRP)

**Fig. 4:**
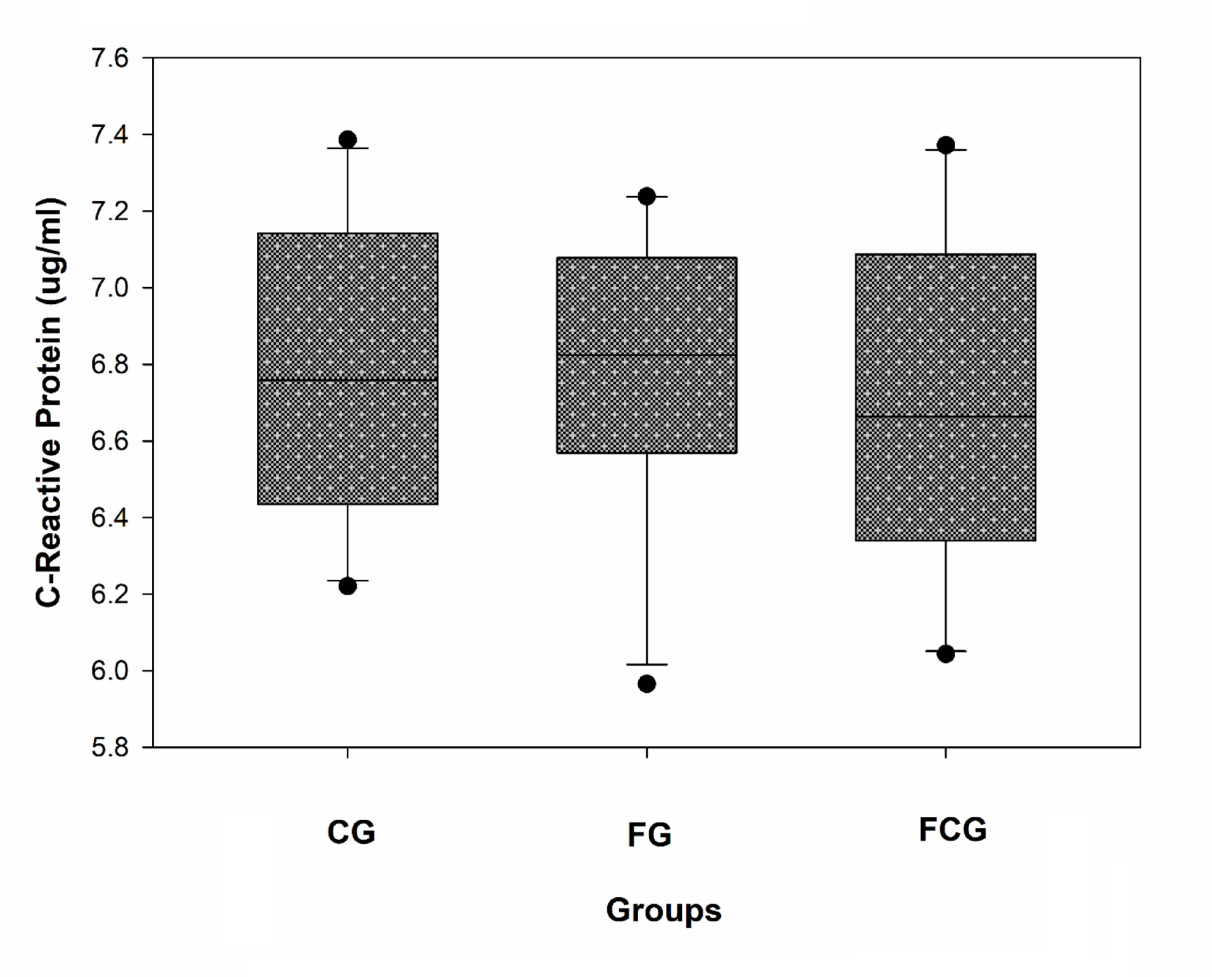
Effect of CCJ12 on C-reactive protein. Plasma C-reactive protein was measured as described in “Materials and Methods”. CG, a control diet; FG, a high fat diet, FCG, a high fat diet with CCJ12. The data was analyzed using one-way ANOVA with Holm-Sidak method and the difference of CRP levels was found insignificant between the groups. Data is detonated as the mean ± SD (n = 10).

CRP is not only a cardiovascular risk factor but also an inflammatory risk factor^39^. Therefore, the effect of CCJ12 on CRP was investigated herein. CRP levels were not significantly different in all three groups, particularly between the FG and FCG groups (Fig. 4). This data suggests that CCJ12 may not have any significant effect on CRP in the FCG group, in comparison to the FG group.

#### Effects of CCJ12 on sE-selectin

**Fig. 5:**
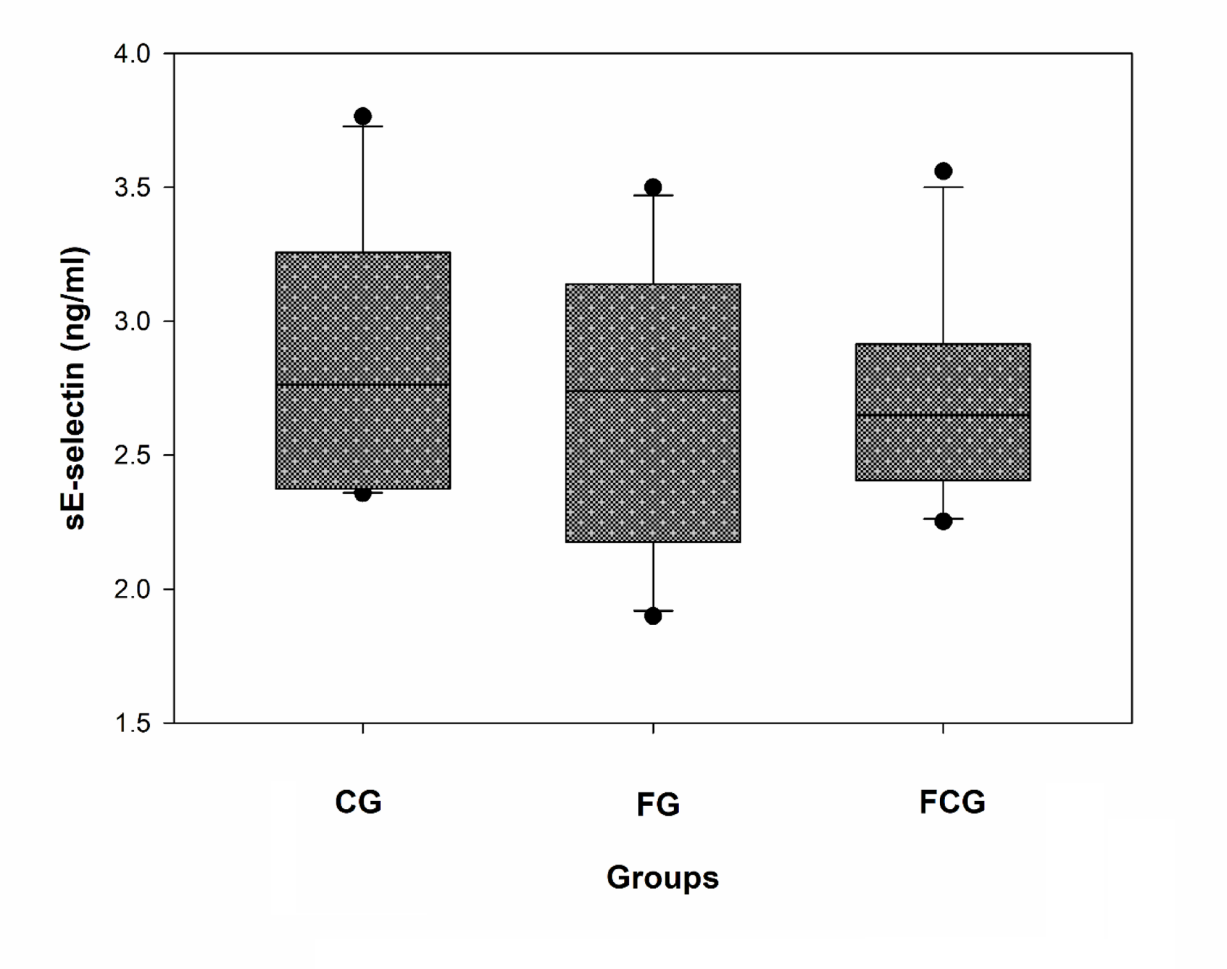
Effect of CCJ12 on sE-selectin. Plasma sE-selectin was measured as described in “Materials and Methods”. CG, a control diet; FG, a high fat diet, FCG, a high fat diet with CCJ12. The data was analyzed using one-way ANOVA with Holm-Sidak method and the difference of sE-selectin levels was found insignificant between the groups. Data is detonated as the mean ± SD (n = 10).

The effect of CCJ12 on sE-selectin expression was examined in the samples, because sE-selectin is a good cardiovascular risk factor^40^. The data showed no significant difference in sE-selectin level between the three groups, especially between the FG and FCG groups (Fig. 5). The data of CRP and sE-selectin indicates that CCJ12 may not have significant effects on sE-selectin and CRP in the FCG group, in comparison to the FG group.

#### Effects of CCJ12 on MCP-1 and TNF-alpha

**Fig. 6:**
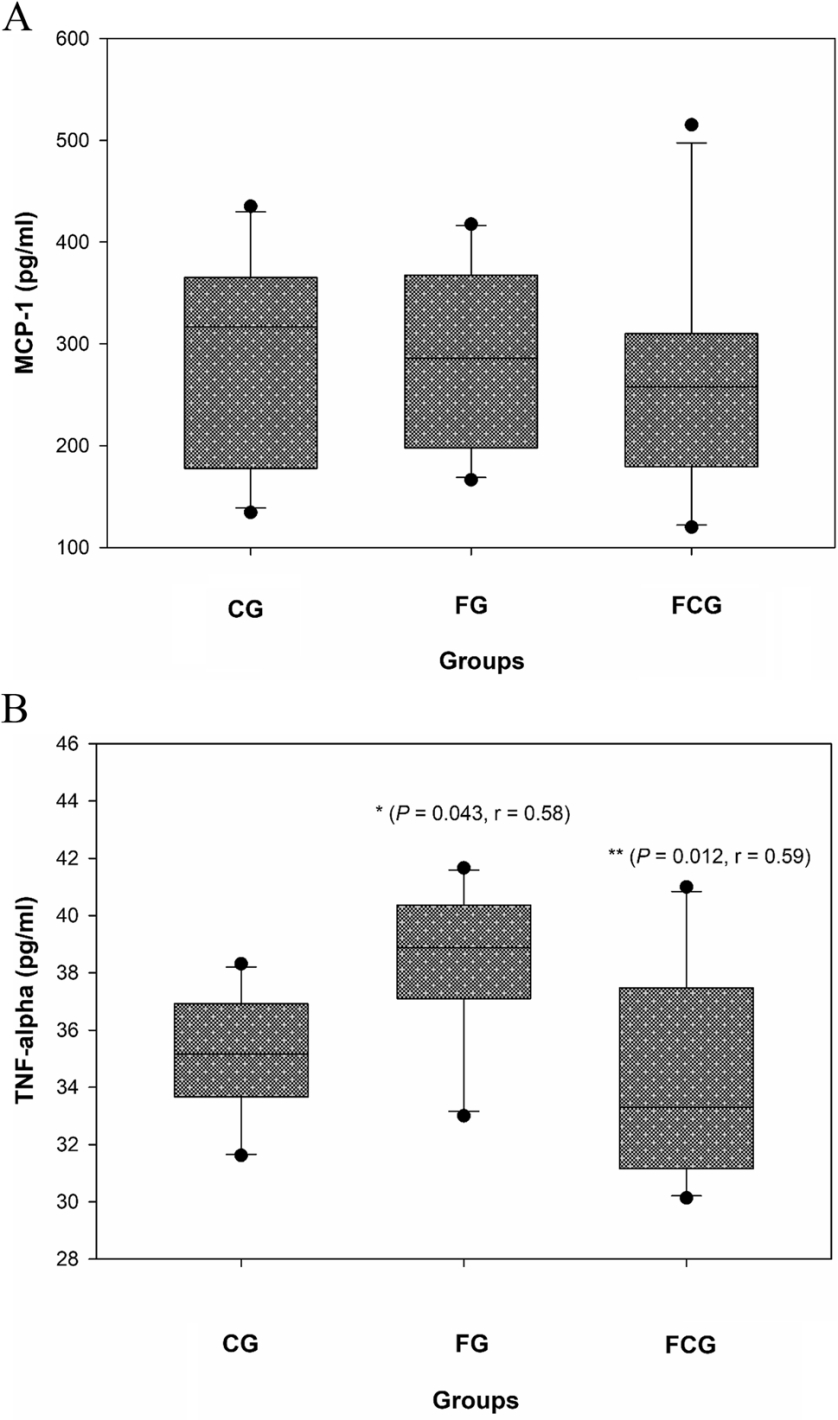
Effects of CCJ12 on MCP-1 and TNF-alpha. MCP-1 (**A**) and TNF-alpha (**B**) were measured using rat plasma samples. CG, a control diet; FG, a high fat diet, FCG, a high fat diet with CCJ12. Data was analyzed using the one-way ANOVA with Holm-Sidak method. Data is detonated as the mean ± SD (n = 10), r is effect size, the mark (*) indicates statistical significance compared to the CG group (*P* < 0.05), and the mark (**) indicates statistical significance compared to the FG group (*P* < 0.05).

Potential effects of CCJ12 on MCP-1 and TNF-alpha were studied because these inflammatory cytokines are associated with the development and progression of many chronic/acute diseases as well as obesity^41–45^. As shown in Fig. 6A, the plasma level of MCP-1 showed no significant difference between the CG, FG and FCG groups. Although a small decrease of MCP-1 was noticed in the FCG, it was deemed non-significant. In contrast to MCP-1, there was a significant increase of TNF-alpha level in the FG group, in comparison to the CG group (*P* < 0.05), suggesting that the level of TNF-alpha may be up in rats fed a high-fat diet, in comparison to rats fed a control diet. However, the level of TNF-alpha was found down in the FCG group, in comparison to the FG group (*P* < 0.05), indicating that CCJ12 may have some positive effect on TNF-alpha in the FCG group, in comparison to the FG group. Again, the outcomes were similar even if the Turkey or Bonferroni methods were used for analysis. Altogether, the data suggests that CCJ12 may have no adverse effects on bodyweight, cholesterol, HDL, LDL, leptin, adiponectin, sE-selectin, CRP, and MCP-1, but have a positive effect on TNF-alpha in rats fed a high fat diet.

## Discussion

Coffee is known to contain many bioactive chemicals including caffeine, chlorogenic acids, and javamide I/II^24–26,35^. However, their amounts in coffee beans can be altered by many factors including the changes of weather and cultivation conditions. In fact, weather change is believed to have significant effects on both the cultivation and chemical composition of Arabica and Robusta beans^22,23^. Furthermore, as temperature increases, the cultivation of Robusta beans has more advantages than that of Arabica beans^22,23^. Javamide I/II are phenolic-conjugated tryptophan compounds found in coffee beans and products, and their amounts are reported to be higher in Robusta beans than Arabica beans. Therefore, more coffee products containing javamide I/II are likely to be found in the market. In fact, several studies already reported that coffee products containing javamide I/II can be commonly available in the market^24–26,35^. However, there is currently limited information about the effects of coffee containing javamide I/II (CCJ12) on obesity, which is considered a multifactorial disease. Therefore, potential effects of CCJ12 on various obesity-related risk factors (bodyweight, cholesterol, HDL, LDL, and triglycerides, leptin, adiponectin, sE-selectin, CRP, MCP-1 and TNF-alpha) were studied in rats fed a high fat diet. Especially, in this study, CCJ12 was analyzed using CIL/LC–MS to provide an additional reference of coffee compounds beyond major coffee compounds (e.g., caffeine, chlorogenic acids, javamide I/II), because CIL/LC–MS is a high throughput approach able to detect a wide range of components with high precision and accuracy. As shown in Table S1, > 700 including several known coffee compounds were identified in CCJ12 by CIL/LC–MS (Table 1). Although CIL/LC–MS has a great advantage in analyzing a wide range of coffee compounds, this method alone could not quantify the amounts of specific coffee compounds. Therefore, in this study, HPLC was used to provide the amounts of major coffee chemicals (e.g., caffeine, javamide I/II, and chlorogenic acids) in CCJ12.

Using CCJ12, in vivo effects on bodyweight, cholesterol, HDL, LDL, and triglycerides, leptin, adiponectin, sE-selectin, CRP, MCP-1 and TNF-alpha factors were studied in a high fat diet-induced obesity rodent model. During the study, bodyweights of rats were monitored, and it was found that a steady increase of bodyweight occurred in rats (from 10- to 30-week-old). Also, water intake was monitored during the study (Fig. 1), because CCJ12 was supplied in drinking water. Using the data, the daily intakes of javamide I/II, caffeine and chlorogenic acids were calculated (Fig. 1). This data provided critical information about the daily intakes of these coffee chemicals, which are often missing in many coffee studies^16–21^. As shown in Fig. 1, the average water intakes of the FG and FCG group were not significantly different, indicating that CCJ12 may have little impact on the water intake in rats fed a high fat diet. Similarly, the average food intake was similar between all three groups, especially between the FG and FCG groups (Fig. 2), suggesting that CCJ12 may not cause craving or avoidance for food. This data suggests that CCJ12 may have little effects on the intakes of water and food in the FCG group, in comparison to the FG group. However, the animals in the FG and FCG groups gained more bodyweight in comparison to the CG group, likely due to a high fat diet (Fig. 2). As expected, the FG and FCG groups showed different levels of HDL, LDL, and TG, in comparison to the CG group (Table 2). However, there were no significant differences in bodyweight, HDL, LDL and TG between the FG and FCG groups. Like metabolic factors, adiponectin and leptin are also associated with increasing obesity and other diseases^47–51^. Therefore, the effects of CCJ12 on adiponectin and leptin were studied. The data showed higher leptin levels in the FG and FCG groups, in comparison to the CG group (Fig. 3). However, no significant difference was found between the FG and FCG groups. Unlike leptin, no significant difference was found in the level of adiponectin between all three groups. Particularly, no significant difference was found in the level of adiponectin between the FG and FCG groups. Likewise, no significant difference was found in plasma CRP level between all three groups, although the FCG group did showed a non-significant reduction, compared to the FG group (Fig. 4). This data suggests that CCJ12 may have no significant effect on CRP in the FCG group, in comparison to the FG group. In fact, this data is in line with a previous reported systematic meta-analysis data that there may be no statistically significant associations of CRP among coffee drinkers^39^. E-selectin is also a well-known biomarker for atherosclerosis^40^. Therefore, the effect of CCJ12 on sE-selectin was studied in this study. CCJ12 was found to have no significant effect on sE-selectin level in the FCG group, in comparison to the FG group (Fig. 5).

In the obese, inflammatory cytokines such as MCP-1 and TNF-alpha have been reported to be potential risk factors for years^42–45^. Specifically, TNF-alpha is a major pro-inflammatory cytokine produced from a variety of cells (e.g., monocytes, macrophages, endothelial cells, adipose tissue), and associated with many inflammatory diseases including diabetes and cardiovascular diseases^41–43^. Like TNF-alpha, MCP-1 is a cytokine produced from macrophages and other cells to attract monocytes, dendritic cells, and T cells to the sites of inflamed regions. Increased MCP-1 expression is also associated with obesity and other diseases^44,45^. Therefore, potential effects of CCJ12 on MCP-1 and TNF-alpha were studied in this paper. The average level of MCP-1 was found to be lower in the FCG group, in comparison to the FG group (Fig. 6A). However, the difference was non-significant. Despite this, this data implies that coffee containing higher amounts javamide I/II may have potential to lower MCP-1, because our previous data showed that javamide I/II can inhibit the expression of inflammatory cytokines in PBMCs^35^. Related to TNF-alpha, the level was found to be lower in the FCG group than the FG group, suggesting that CCJ12 may have a positive effect on TNF-alpha in the FCG group (Fig. 6B). In fact, there are reports about health effects of coffee on TNF-alpha and other factors^52,53^. Particularly, chlorogenic acids were reported to have several positive effects such as anti-inflammatory, anti-diabetic, and anti-cancer effects^16,52,53^. However, the amounts of javamide I/II were not determined in the studies, leaving a question about the potential contribution of javamide-I/-II on the reported beneficial coffee effects^16,17,52,53^. Therefore, future studies using CCJ12 with higher amounts of javamide I/II should be conducted to examine potential roles of javamide I/II on inflammatory cytokines and other underlined mechanisms. Altogether, based on a previous study^34^ and this study, CCJ12 may not have any adverse effects on people with normal weight and obesity. However, more studies including human subjects may be needed to determine the benefits and drawbacks of coffee consumption related to obesity and other health conditions because obesity is notably associated with many chronic diseases. Furthermore, in the future, potential effects of javamide I/II themselves on metabolic, cardiovascular, inflammatory and other factors should be investigated for the comparison to the effects of CCJ12.

## Conclusion

In CCJ12, > 700 compounds were identified by LC/MS, and caffeine, javamide I/II, and chlorogenic acids were quantified by HPLC. The data of animal study showed no significant discrepancy in water/food intakes between all groups. However, the FG and FCG groups gained more weight, in comparison to the CG group, likely due to a high-fat diet (*P* < 0.05). Consequently, the FG and FCG groups showed higher levels of LDL, TG, and leptin than the CG group (*P* < 0.05). However, no significant differences were found in bodyweight, LDL, HDL, TG, leptin, and adiponectin levels between the FG and FCG groups. Also, no significant differences were found in sE-selectin, C-reactive protein, and MCP-1 levels between the FG and FCG groups. However, the data showed that TNF-alpha levels were down in the FCG group in comparison to the FG group (*P* < 0.05), suggesting that CCJ12 may have a positive effect on TNF-alpha in the FCG group. Altogether, CCJ12 may have a positive health effect on inflammatory cytokine TNF-alpha without adverse effects on bodyweight, HDL, LDL, TG, leptin, adiponectin, sE-selectin, C-reactive protein, and MCP-1 in rats fed a high fat diet.

## Supplementary Information

Below is the link to the electronic supplementary material.

Supplementary Material 1

## Data Availability

"All data/tables/figures/supplement data mentioned in this study are included in the article/supplementary material."
