# Supplementary material for: Compositional analysis of coffee containing javamide I/II and investigation of health effects in rats fed a high fat diet
Source: Sci Rep. 2025 Aug 6;15:28806. doi: 10.1038/s41598-025-13590-3 (PMC12328587; doi:10.1038/s41598-025-13590-3)
Supplement: Supplementary file 1 — Supplementary Material 1 [file 41598_2025_13590_MOESM1_ESM.pdf]

Supplement data

Table S1

The list of compounds found in coffee containing javamide I/II (CCJ12). All compounds were identified using CIL-LC/MS (chemical isotope-labelling-LC/MS) as described in “Materials and Methods”. Mass (Da) represents the molecular weight of each compound, and Pk detonates the peak of each compound.

Mass (Da)	Compound Name	Relative Pk signal
31.0405	Methylamine	1.10825
59.038	Aminoacetaldehyde	0.513
59.0479	Guanidine	0.48375
60.0212	Acetic acid	2.9315
61.053	Ethanolamine	0.1365
62.0004	Carbonic acid	1.7085
71.0728	Pyrrolidine	1.45375
73.0514	Aminoacetone	0.73525
73.0883	Diethylamine	0.844
73.0884	Isomer 1 of Diethylamine	0.51875
74.0471	Acetylhydrazine	0.39825
74.0842	1,3-Diaminopropane	0.86125
75.0314	Glycine	0.101
75.0315	Isomer 1 of Glycine	0.9225
75.0674	Isomer 1 of (R)-1-Aminopropan-2-ol	0.85775
75.0676	(R)-1-Aminopropan-2-ol	2.04125
76.0159	Glycolic acid	1.48525
85.0518	Gamma-Aminobutyric acid	0.013
85.0524	3-Aminoisobutanoic acid	0.316425
86.0366	Isomer 1 of Crotonic Acid	1.118
86.0367	Crotonic Acid	2.94875
87.0316	Aminoacrylic acid	0.2245
87.0678	4-Aminobutyraldehyde	0.24025

87.0678	Isomer 1 of Morpholine	0.939
87.0679	Isomer 1 of 4-Aminobutyraldehyde	0.9005
87.0679	Morpholine	0.8855
87.0683	Isomer 2 of Morpholine	1.21075
88.0523	Butanoic Acid	3.24375
88.0998	1,4-diaminobutane	0.005
89.0108	Oxamic acid	0.275
89.0461	Alanine	0.10675
89.0474	Sarcosine	0.34575
89.0489	Beta-Alanine	0.3725
89.995	Oxalic Acid	0.6375
89.9955	Isomer 1 of Oxalic Acid	1.44175
90.0302	Methoxyacetic acid	2.2705
90.031	(S)-Lactic acid / (R)-Lactic acid	2.3955
90.0312	Hydracrylic Acid	1.21275
90.0316	Monoethyl carbonic acid	2.99725
90.0319	Isomer 1 of Monoethyl carbonic acid	1.705
95.0371	2-Hydroxypyridine	4.028
100.0522	3-Methylcrotonic Acid	1.5635
100.0522	Tiglic Acid	1.6095
101.0473	Isomer 1 of Homoserine	0.316
101.0476	(S)-2-Azetidinecarboxylic acid	0.7445
101.0832	Isomer 1 of 4-Hydroxypiperidine	1.4875
101.0833	5-Aminopentanal	0.76275
101.0836	4-Hydroxypiperidine	0.104
101.1207	Hexylamine	1.13425
102.0313	Succinic Semialdehyde	0.594
102.0317	Isomer 1 of Alpha-Ketobutyric Acid	0.239
102.0321	Alpha-Ketobutyric Acid	0.4415

102.0676	Iso-Valeric Acid	2.0285
103.0622	Isomer 1 of 3-Aminoisobutanoic acid	0.41925
103.0626	N-Ethylglycine	0.86325
103.0627	3-Aminobutanoic acid	0.80625
103.0629	Isomer 1 of N-Ethylglycine	0.437
103.063	Alpha-aminobutyric acid	0.495
103.0994	Choline	0.33125
104.0109	Hydroxypyruvic acid	3.3155
104.011	Isomer 1 of Malonic Acid	0.69475
104.0111	Isomer 2 of Malonic Acid	1.67075
104.0113	Malonic Acid	0.28175
104.0467	2-Hydroxybutyric Acid	4.93125
105.0424	Serine	0.059
106.026	Glyceric Acid	1.39525
106.0262	Isomer 1 of Glyceric Acid	0.3585
106.0262	Isomer 2 of Glyceric Acid	0.728
109.0532	Hydroxylaminobenzene	4.73675
109.0538	2-Aminophenol	0.4785
110.036	Isomer 1 of Resorcinol	0.61875
110.0361	Resorcinol	0.24775
110.0366	Catechol	0.6755
110.0369	Isomer 3 of Resorcinol	0.80025
110.0369	Isomer 4 of Resorcinol	0.73025
110.037	Isomer 2 of Resorcinol	0.71175
110.0389	Hydroquinone	0.07975
111.0316	Pyrrole-2-Carboxylic Acid	1.47175
111.0321	2,6-Dihydroxypyridine	0.74125
112.0161	3-Furoic Acid	3.5315
112.0268	Uracil	2.25525

112.0269	4-Carboxypyrazole	0.1175
114.0314	2-Hydroxy-2,4-pentadienoic acid	1.1655
114.0316	Isomer 1 of 2-Hydroxy-2,4-pentadienoic acid	0.712
114.0317	Norfuraneol	0.274
114.0321	Isomer 1 of Norfuraneol	0.54675
114.0423	Isomer 1 of 3-Cyano-L-alanine	0.28125
114.0428	3-Cyano-L-alanine	0.06425
114.079	3-Amino-2-piperidone	0.19975
115.0266	Maleamic acid	0.4775
115.0625	Proline	0.0545
116.0106	Isomer 1 of Fumaric Acid	1.1785
116.0114	Isomer 2 of Fumaric Acid	0.25925
116.0115	Fumaric Acid	0.19275
116.0467	5-Oxopentanoic acid	1.25025
116.0468	Tetrahydro-2-Furoic Acid	4.2025
116.0469	Levulinic Acid	3.5595
117.0422	N-Acetyl-Glycine	1.36025
117.0425	L-Aspartate 4-semialdehyde	1.0965
117.0442	L-2-Amino-3-oxobutanoic acid	2.22925
117.0774	4-Methylaminobutyric acid	0.3005
117.0782	Isomer 2 of 5-Aminopentanoic acid	0.95325
117.0782	Valine	0.07375
117.0785	N-Methyl-A-Aminoisobutyric Acid	1.91175
117.0786	5-Aminopentanoic acid	0.4515
117.0803	Isomer 1 of 5-Aminopentanoic acid	1.31325
118.0262	Isomer 1 of Succinic Acid	1.17925
118.0262	Methylmalonic acid	4.036
118.0263	Isomer 1 of Methylmalonic acid	0.67725
118.0271	Succinic Acid	0.2695

118.0611	3-Hydroxyisovaleric Acid	0.64675
118.0731	2,4-Diaminobutyric acid	0.203
119.0212	Aminomalonic acid	0.14975
119.0577	Threonine	0.16125
119.0579	Isomer 2 of Threonine	0.24675
119.0581	Homoserine	0.22675
119.0586	Isomer 1 of Threonine	0.30125
120.0422	4-Deoxyerythronic acid	2.1785
120.0562	4-Hydroxystyrene	2.5155
121.0891	2,6-Dimethylaniline	0.2762
122.037	Benzoic Acid	1.35425
123.0316	Picolinic Acid	4.3415
123.0692	p-Anisidine	3.981
124.052	2,3-Dihydroxytoluene	4.894
124.0531	4-Hydroxybenzyl alcohol	1.4555
125.0469	4-Aminocatechol	0.5285
125.0472	3-Hydroxyaminophenol	2.24325
125.048	Isomer 1 of 3-Hydroxyaminophenol	2.8375
125.0487	Aminohydroquinone	1.192
126.0332	1,2,3-Trihydroxybenzene	0.3985
127.063	(S)-2,3,4,5-Tetrahydropyridine-2-carboxylic acid	0.0681
128.0472	2-Hydroxy-cis-hex-2,4-dienoic acid	3.123
128.0472	3-Hydroxy-4,5-dimethyl-2(5H)-furanone	2.1025
128.0581	gamma-Amino-gamma-cyanobutanoic acid	0.24075
129.0421	Glutamic Acid	0.00475
129.0421	L-beta-Ethynylserine	0.91225
129.0422	Pyroglutamic Acid	1.38275
129.0424	L-1-Pyrroline-3-hydroxy-5-carboxylic acid	0.386
129.0786	Pipecolic acid	0.033

129.0787	2-Pyrrolidineacetic acid	0.64925
130.0262	Isomer 1 of Mesaconic Acid	3.2515
130.0263	Isomer 2 of Mesaconic Acid	3.75975
130.0264	Isomer 2 of Trans-Glutaconic Acid	4.03125
130.0265	Mesaconic Acid	0.70975
130.0266	Trans-Glutaconic Acid	1.07025
130.0271	2,5-Dioxopentanoic acid	3.5415
130.0271	Isomer 1 of Itaconic acid	0.48425
130.0278	Isomer 3 of Citraconic Acid	0.5325
130.0377	Ureidoacrylic acid	0.87
130.0626	4-Methyl-2-Oxopentanoic Acid	0.122
130.0627	Adipate semialdehyde	3.157
130.0629	2-Ketohexanoic Acid	0.0555
130.1087	Isomer 2 of N-Acetylputrescine	0.30075
130.1103	Isomer 1 of N-Acetylputrescine	0.2805
130.1123	N-Acetylputrescine	0.168
131.0578	5-Aminolevulinic acid	0.3285
131.0579	4-Hydroxyproline	0.07425
131.0922	Leucine	0.0645
131.0936	Norleucine	0.53125
131.0938	Isomer 1 of 6-Aminohexanoic acid	5.03025
131.0939	E-Aminocaproic Acid	1.28375
131.0944	Isoleucine	0.01425
132.0057	2-Hydroxyethylenedicarboxylic acid	0.502
132.0419	Isomer 1 of Glutaric Acid	2.481
132.042	Isomer 1 of Methylsuccinic Acid	3.84575
132.0421	Dimethylmalonic acid	2.002
132.0422	Methylsuccinic Acid	1.1705
132.0424	Monoethyl malonic acid	4.87575

132.0428	Glutaric Acid	0.603
132.0526	Asparagine	0.0065
132.0531	Glycyl-glycine	0.29075
132.0773	Isomer 1 of 6-Hydroxyhexanoic acid	1.1535
132.0781	6-Hydroxyhexanoic acid	0.59675
132.0789	2-Hydroxycaproic Acid	3.00625
132.091	Ornithine	0.14175
133.0371	Aspartate	0.02525
133.0376	Isomer 1 of Aspartate	0.44075
133.0379	Iminodiacetic acid	0.34725
133.0731	Isomer 1 of L-2-Amino-5-hydroxypentanoic acid	0.44725
133.0732	L-2-Amino-5-hydroxypentanoic acid	0.245
134.0211	Malic Acid	0.3855
134.0223	Isomer 1 of Malic Acid	0.1175
134.0571	2,3-Dihydroxy-3-methylbutanoic acid	1.97475
135.054	Adenine	2.5685
136.0389	Hypoxanthine	3.1625
136.0524	3,4-Dihydroxystyrene	4.63225
136.0525	m-Methylbenzoic acid	0.6245
136.0525	Phenylacetic Acid	1.3215
137.0469	2-Aminobenzoic acid	0.77625
137.0474	2-Pyridylacetic acid	0.95075
137.0475	P-Aminobenzoic Acid	1.04075
137.0476	Isomer 1 of 2-Pyridylacetic acid	0.65575
137.0477	Isomer 1 of 2-Aminobenzoic acid	3.30475
137.0481	m-Aminobenzoic acid	0.253
137.0485	Trigonelline (N-Methylnicotinic Acid)	0.8185
137.0838	Tyramine	0.41675
138.0293	Gentisate aldehyde	1.30125

138.0314	3-Hydroxybenzoic Acid	1.16425
138.0316	Salicylic Acid	2.217
138.032	Isomer 1 of 3-Hydroxybenzoic Acid	2.92725
138.032	Isomer 2 of 3-Hydroxybenzoic Acid	0.314
138.0321	3,4-Dihydroxybenzaldehyde	0.87325
138.0329	Isomer 1 of 3,4-Dihydroxybenzaldehyde	1.089
138.0666	2,6-Dimethyl-1,4-benzenediol	4.2325
138.0674	4-Hydroxyphenylethanol	1.5505
138.0675	Isomer 1 of 4-Hydroxyphenylethanol	2.411
139.0266	3-Hydroxypicolinic acid	3.94825
139.0267	Isomer 1 of 6-Hydroxynicotinic Acid	1.37325
139.0275	6-Hydroxynicotinic Acid	1.897
139.063	3,4-Dihydroxybenzylamine	3.74275
140.0106	Isomer 1 of cis-4-Carboxymethylenebut-2-en-4-olide	2.2795
140.0107	cis-4-Carboxymethylenebut-2-en-4-olide	2.95875
140.0476	4-Hydroxymethylcatechol	1.28725
141.0176	O-Phosphoethanolamine	0.45625
142.0258	(S)-5-Oxo-2,5-dihydrofuran-2-acetic acid	2.02825
142.026	Cis,Cis-Muconic Acid	1.85825
142.0379	5-Hydroxymethyluracil	0.31625
142.0739	Ectoine	1.007
142.0989	2-Octenoic Acid	0.90475
143.0576	Vinylacetylglycine	1.17225
143.0585	Isomer 1 of Vinylacetylglycine	0.467
143.0941	L-2-Amino-3-methylenehexanoic acid	0.07795
143.9961	3-Chlorocatechol	0.031
143.9969	Isomer 1 of 3-Chlorocatechol	0.0755
144.0416	2-Methyleneglutaric acid	3.60075
144.0417	2,3-Dimethylmaleic acid	3.5055

144.0417	3-Hexenedioic acid	3.92275
144.0417	Isomer 1 of 3-Hexenedioic acid	2.094975
144.0423	Isomer 2 of 3-Hexenedioic acid	0.39825
144.0424	Isomer 3 of 3-Hexenedioic acid	0.42875
144.0427	Isomer 1 of 2,3-Dimethylmaleic acid	0.523
144.0521	N-Acetyl-N-Formiminoglycine	0.32525
144.0778	4-Hydroxycyclohexylcarboxylic Acid	0.52275
144.0782	7-Oxoheptanoic acid	2.3195
144.1141	Octanoic acid	2.37525
144.1143	Isomer 1 of Octanoic acid	0.465
144.1381	Homoagmatine	0.65325
145.0369	4-Oxoglutaramic acid	0.37575
145.0723	(S)-5-Amino-3-oxohexanoic acid	0.6655
145.0731	4-Acetamidobutanoic acid	1.53675
145.0731	Isomer 1 of 6-Amino-2-oxohexanoic acid	0.56175
145.0732	N-Acetyl-(S)-2-Aminobutanoic acid	1.034
145.0739	6-Amino-2-oxohexanoic acid	0.1725
146.0213	2-Oxoglutaric acid	0.1105
146.0246	3-Oxoglutaric acid	0.186
146.0562	2,2-Dimethylsuccinic Acid	2.76475
146.0571	(R)-3-Hydroxy-3-methyl-2-oxopentanoic acid	4.95275
146.0578	Methylglutaric Acid	0.7165
146.058	Adipic Acid	0.58975
146.0683	Glutamine	0.19875
146.0691	Alanyl-Glycine	0.1445
146.0939	3-Hydroxyisoheptanoic acid	2.26725
147.052	Isomer 2 of N-Methyl aspartic acid	0.3505
147.0521	N-Acetyl-L-Serine	1.004
147.0523	Isomer 1 of Glutamic Acid	0.1725

147.0525	Isomer 1 of L-threo-3-Methylaspartic acid	0.13625
147.053	L-threo-3-Methylaspartic acid	0.771
147.053	N-Methyl aspartic acid	0.13425
147.0534	O-Acetyl-L-serine	0.06875
147.0544	Isomer 1 of N-Methyl aspartic acid	0.32675
148.0363	L-threo-3-Methylmalic acid	0.7
148.0364	Isomer 1 of 2-Hydroxyglutaric Acid	1.4075
148.0364	Isomer 1 of L-threo-3-Methylmalic acid	0.42325
148.0366	Isomer 2 of L-threo-3-Methylmalic acid	1.70675
148.0372	Isomer 3 of L-threo-3-Methylmalic acid	0.06875
148.0376	2-Hydroxyglutaric Acid	0.3565
148.0377	2-Hydroxy-2-Methylbutanedioic Acid	0.51475
148.0729	(R)-2,3-Dihydroxy-3-methylpentanoic acid	1.45225
149.0697	6-Methyladenine	2.2535
150.0314	Phenylglyoxylic Acid	0.07475
150.0675	4-Ethylbenzoic Acid	0.31075
150.0679	2-Methoxy-4-vinylphenol	3.3325
151.0299	(S)C(S)S-S-Methylcysteine sulfoxide	0.15455
151.0493	8-Hydroxyadenine	1.042
151.0625	2-Phenylglycine	0.30775
151.0625	Isomer 1 of 2-Phenylglycine	3.6685
151.0628	2-Amino-3-methylbenzoic acid	0.40175
151.063	2-(Methylamino)benzoic acid	1.11575
152.0333	Xanthine	0.72575
152.0459	3,4-Dihydroxyphenylacetaldehyde	4.894
152.0461	Isomer 1 of 3,4-Dihydroxyphenylacetaldehyde	0.39425
152.0464	3',5'-Dihydroxyacetophenone	4.87
152.0465	Parahydroxyphenylacetic Acid	2.351
152.0469	Isomer 2 of 3,4-Dihydroxyphenylacetaldehyde	0.31625

152.047	2-Methoxybenzoic acid	2.95365
152.0472	4-Hydroxy-3-Methylbenzoic Acid	4.02875
152.0473	Ortho-Hydroxyphenylacetic acid	1.66975
152.0477	4-Methylsalicylic acid	3.5857
152.0478	3-Hydroxyphenylacetic acid	1.546
153.0089	3-Sulfino-L-alanine	0.08225
153.0424	3-Hydroxy-2-methylpyridine-5-carboxylic acid	1.7315
153.0427	3-Hydroxyanthranilic acid	1.125
153.0429	o-Hydroxylaminobenzoic acid	2.84775
154.0252	Protocatechuic acid	2.98475
154.0266	Gentisic acid	4.87325
154.0993	3,5,5-Trimethyl-1,2-cyclohexanedione	4.3425
155.021	2,6-Dihydroxynicotinic acid	0.1405
156.0064	2,5-Furandicarboxylic acid	0.33325
156.0418	(1R,6S)-1,6-Dihydroxycyclohexa-2,4-diene-1-carboxylic acid	1.472625
156.0784	8-Hydroxy-5,6-octadienoic acid	2.98425
157.0369	2-Aminomuconic acid	0.5085
157.0733	3-Methylcrotonylglycine	2.0095
157.0733	N-Acetyl-L-Proline	1.569
158.0215	Zymonic acid	0.28125
158.0569	Succinylacetone	2.443
158.0577	2-Isopropylmaleic acid	0.8275
158.0937	2-n-Propyl-4-oxopentanoic acid	1.579
159.0527	4-Methylene-L-glutamic acid	0.2525
159.0528	N-Acetyl-L-2-Amino-3-oxobutanoic acid	1.24125
159.0889	N-Isovaleroylglycine	1.82175
159.0894	Calystegin A3	0.248
159.1257	2-Aminooctanoic acid	0.38875
160.0366	3-Oxoadipic acid	1.4565

160.0732	3-Methyladipic Acid	0.8155	
160.0847	N(alpha)-Acetyl-L-2,4-diaminobutyric acid	0.1785	
160.085	Alanyl-Alanine	0.17325	
160.0998	Tryptamine	0.2045	
160.1093	Hydroxyoctanoic Acid	1.10475	
160.1096	Hydroxyoctanoic acid	0.92275	
160.1101	Isomer 1 of Hydroxyoctanoic Acid	0.83975	
160.1197	N(6)-Methyllysine	0.57075	
161.0307	Isomer 2 of A-Ketoglutaric acid oxime	0.17125	
161.0321	Isomer 1 of A-Ketoglutaric acid oxime	0.217	
161.0326	A-Ketoglutaric acid oxime	0.22275	
161.0681	O-Acetyl-L-homoserine	0.797	
161.0686	4-Methyl-L-glutamic acid	0.07525	
161.0687	N-Methyl-L-glutamic acid	0.108	
161.0691	Aminoadipic acid	0.137	
162.0302	Isomer 2 of 1,2-Dihydroxy-5-(methylthio)pent-1-en-3-one		1.88575
162.0313	Isomer 1 of 1,2-Dihydroxy-5-(methylthio)pent-1-en-3-one		1.61175
162.0313	Isomer 7 of 1,2-Dihydroxy-5-(methylthio)pent-1-en-3-one		0.096
162.0315	1,2-Dihydroxy-5-(methylthio)pent-1-en-3-one	0.2665	
162.0315	Isomer 3 of 1,2-Dihydroxy-5-(methylthio)pent-1-en-3-one		1.62425
162.0317	Umbelliferone	1.90725	
162.0318	Isomer 5 of 1,2-Dihydroxy-5-(methylthio)pent-1-en-3-one		0.40375
162.0319	2-Oxo-5-methylthiopentanoic acid	0.164	
162.035	Isomer 4 of 1,2-Dihydroxy-5-(methylthio)pent-1-en-3-one		0.752
162.0382	Isomer 6 of 1,2-Dihydroxy-5-(methylthio)pent-1-en-3-one		0.41325
162.0517	Isomer 1 of 3-Ethylmalic acid	1.16675	
162.0518	3-Ethylmalic acid	0.4115	
162.0519	(R)-2-Ethylmalic acid	2.01575	
162.052	3-Hydroxyadipic acid	1.45425	

162.0522	(R)-3,3-Dimethylmalic acid	3.14775
162.0527	3-Hydroxymethylglutaric Acid	0.2445
162.0528	(S)-2-(Hydroxymethyl)glutaric acid	0.2075
162.0994	5-Hydroxylysine	0.13275
163.0624	4-(3-Pyridyl)-3-butenoic acid	1.7215
163.0628	Homomethionine	0.20775
163.0831	Isomer 1 of 2-Deoxy-scyllo-inosamine	0.21345
163.0838	2-Deoxy-scyllo-inosamine	0.1745
164.0456	Phenylpyruvic Acid	0.714
164.0459	Caffeic aldehyde	4.1665
164.0464	Isomer 1 of Caffeic aldehyde	0.2105
164.0473	m-Coumaric acid	0.52625
165.0422	Formylanthranilic acid	1.80725
165.0464	Methionine Sulfoxide	0.071
165.0649	1-Methylguanine	1.6805
165.0772	L-Phenylalanine / D-Phenylalanine	1.73825
166.0257	Phthalic Acid	1.2405
166.0262	Terephthalic Acid	0.3235
166.0607	3,4-Dihydroxyphenylacetone	4.894
166.0631	Desaminotyrosine	0.6625
167.0575	3-Methoxyanthranilic acid	0.32
167.0578	Noradrenochrome o-semiquinone	2.286
167.0579	L-4-Hydroxyphenylglycine	1.28225
167.0937	Phenylephrine	0.162
168.041	Homogentisic acid	4.642
168.042	Isomer 1 of 5-Methoxysalicylic Acid	1.3075
168.0423	5-Methoxysalicylic Acid	2.06775
168.0424	Vanillic acid	1.59475
168.0429	2',4',6'-Trihydroxyacetophenone	4.23375

168.1138	Geranic acid	1.11175
169.0737	Pyridoxine	2.3325
169.0847	1-Methylhistidine	0.21275
170.0187	Gallic Acid	0.44825
170.0215	2,3,4-Trihydroxbenzoic acid	4.894
170.0575	3,4-Dihydroxyphenylethyleneglycol	1.6875
170.0577	1,2-Dihydroxy-6-methylcyclohexa-3,5-dienecarboxylic acid	1.90315
171.0525	2,3,4,5-Tetrahydrodipicolinic acid	1.78825
172.073	2-Octenedioic acid	1.0055
172.073	Isomer 1 of 2-Octenedioic acid	0.84
172.0838	Prolyl-Glycine	1.226
172.086	Glycyl-Proline	1.01775
172.1095	9-Oxononanoic acid	0.493
172.1097	Isomer 1 of 9-Oxononanoic acid	0.7245
173.0678	N-Acetyl-L-glutamate 5-semialdehyde	1.6705
173.1048	Hexanoylglycine	1.9485
174.0162	Isomer 2 of Aconitic Acid	0.14925
174.0163	Isomer 1 of Aconitic Acid	0.57975
174.0164	Aconitic Acid	0.0435
174.0518	2-Oxopimelic acid	3.207
174.0523	Shikimic acid	1.64925
174.0527	2-Methyl-3-oxoadipic acid	0.70375
174.0528	Isomer 1 of 2-Methyl-3-oxoadipic acid	0.90775
174.0987	Valyl-Glycine	1.04225
174.0995	Glycyl-Valine	0.3905
174.1002	N5-Acetyl-L-Ornithine	0.37
174.1113	Arginine	0.10275
174.1255	3-Hydroxynonanoic acid	0.66675
175.0491	N-Acetyl-L-aspartic acid	0.36525

175.0631	Indoleacetic Acid	0.54125
175.0997	Citrulline	0.182
176.0317	Parapyruvic acid	0.50175
176.0323	Isomer 1 of Parapyruvic acid	0.2145
178.099	5-Phenylvaleric acid	1.131
180.0403	Isomer 3 of 3-(3,5-Dihydroxyphenyl)-2-propenoic acid	0.24075
180.0406	Isomer 4 of 3-(3,5-Dihydroxyphenyl)-2-propenoic acid	0.24175
180.0407	Isomer 1 of 3-(3,5-Dihydroxyphenyl)-2-propenoic acid	0.21725
180.0411	Isomer 2 of 3-(3,5-Dihydroxyphenyl)-2-propenoic acid	0.25225
180.0416	3-(3,5-Dihydroxyphenyl)-2-propenoic acid	0.364
180.0416	trans-2,3-Dihydroxycinnamic acid	1.702
180.0419	Caffeic Acid	0.214
180.0421	2-Hydroxy-3-(4-hydroxyphenyl)propenoic acid	0.19125
180.0426	Isomer 1 of 2-Hydroxy-3-(4-hydroxyphenyl)propenoic acid	1.12375
180.0622	Isomer 6 of 4-Chloro-L-lysine	1.5885
180.0624	4-Chloro-L-lysine	0.39075
180.0648	Theophylline	0.90075
180.0662	Isomer 3 of 4-Chloro-L-lysine	0.2445
180.0862	5-Hydroxykynurenamine	0.18175
181.0729	Isomer 1 of Tyrosine	1.01675
181.0735	o-Tyrosine	0.96925
181.0737	Tyrosine	0.14525
181.0738	N-Hydroxy-L-phenylalanine	1.59225
182.021	Isomer 1 of 2-Hydroxyisophthalic acid	3.414
182.0215	4-Hydroxyisophthalic acid	2.139
182.0217	2-Hydroxyisophthalic acid	0.3205
182.0564	3-(2,3-Dihydroxyphenyl)propanoic acid	4.65225
182.0574	2,6-Dimethoxybenzoic acid	3.8325
183.0529	4-Pyridoxic acid	3.67925

184.0734	3-Methoxy-4-hydroxyphenylethyleneglycol	3.5125
186.016	3-Carboxy-cis,cis-muconic acid	0.22325
186.0163	2-Carboxy-2,5-dihydro-5-oxofuran-2-acetic acid	0.06475
186.0168	4-Carboxy-2-hydroxymuconate semialdehyde	0.08075
186.0869	Isomer 1 of cis-2-Carboxycyclohexyl-acetic acid	0.5135
186.0884	cis-2-Carboxycyclohexyl-acetic acid	0.3815
186.1003	Alanyl-Proline	1.10625
186.1233	(3S)-6-Hydroxy-3-isopropenyl-heptanoic acid	0.75275
187.0479	1-(Malonylamino)cyclopropanecarboxylic acid	1.517
187.0479	Isomer 2 of 1-(Malonylamino)cyclopropanecarboxylic acid	0.19775
187.0485	Isomer 1 of 1-(Malonylamino)cyclopropanecarboxylic acid	0.26325
188.0317	Isomer 1 of (Z)-But-1-ene-1,2,4-tricarboxylic acid	0.55875
188.0322	(Z)-But-1-ene-1,2,4-tricarboxylic acid	0.20675
188.0679	2-Oxosuberic acid	0.61175
188.1043	Isomer 2 of Azelaic Acid	0.413
188.1046	Azelaic Acid	0.35725
188.1046	Isomer 1 of Azelaic Acid	1.3845
188.1151	Alanyl-Valine	0.15125
188.1156	Glycyl-Leucine	0.211
188.1161	N6-Acetyl-Lysine	0.10875
188.1162	Leucyl-Glycine	0.1845
188.1162	Valyl-Alanine	0.29125
188.1272	Homoarginine	0.20275
188.1409	2-Hydroxydecanoic acid	0.4875
189.064	L-2-Amino-6-oxoheptanedioic acid	0.26425
189.0658	N-Acetyl-L-glutamic acid	0.152
189.0781	Glycyl-Asparagine	1.369
189.1113	Homocitrulline	0.28975
190.0586	L-beta-aspartyl-L-glycine	0.06025

190.0821	3-Hydroxysuberic acid	0.15725
190.0951	2-Amino-4-[(2-hydroxy-1-oxopropyl)amino]butanoic acid	0.21575
191.0429	gamma-Carboxyglutamic acid	0.38725
192.0265	Citric Acid / Isocitric Acid	0.44025
192.0274	Isomer 1 of Citric Acid / Isocitric Acid	0.5025
192.0419	2-Hydroxychromene-2-carboxylic acid	0.16575
192.0425	trans-o-Hydroxybenzylidenepyruvic acid	4.56325
192.044	Scopoletin	1.2205
192.0628	Isomer 3 of Quinic Acid	0.7225
192.063	Isomer 2 of Quinic Acid	1.85325
192.0631	Isomer 1 of Quinic Acid	1.75025
192.0631	Quinic Acid	1.03225
193.0732	p-Methylhippuric Acid	1.007
193.0735	Phenylacetylglycine	0.7945
194.0413	2-Keto-D-gluconic acid / 2-Keto-L-gluconic acid	0.185725
194.0576	5-Hydroxyconiferaldehyde	4.021
194.0587	trans-Ferulic acid	0.28175
194.0941	Isomer 1 of 2,5-Dimethoxy-4-(2-propenyl)phenol	0.397275
194.0948	2,5-Dimethoxy-4-(2-propenyl)phenol	1.5605
194.0984	Zingerone	0.39475
195.074	2-Amino-2-deoxy-D-gluconic acid	0.2525
196.0365	Isomer 2 of 3-(3,4-Dihydroxyphenyl)pyruvic acid	3.64
196.0371	3-(3,4-Dihydroxyphenyl)pyruvic acid	1.368
196.0376	Isomer 1 of 3-(3,4-Dihydroxyphenyl)pyruvic acid	1.18125
196.0376	Isomer 4 of 3-(3,4-Dihydroxyphenyl)pyruvic acid	0.9755
196.0394	Isomer 3 of 3-(3,4-Dihydroxyphenyl)pyruvic acid	1.5365
196.0722	3-(3,4-Dihydroxyphenyl)-2-methylpropionic acid	2.3535
196.0738	Homoveratric acid	2.181
197.0688	3,4-Dihydroxy-L-phenylalanine	0.33025

198.052	Vanillylmandelic acid	0.98225
198.0525	Isomer 2 of 3-(3,4-Dihydroxyphenyl)lactic acid	1.23475
198.0526	3-(3,4-Dihydroxyphenyl)lactic acid	0.23775
198.0534	Syringic acid	1.0035
198.0545	Isomer 1 of 3-(3,4-Dihydroxyphenyl)lactic acid	0.826
198.1612	5-Dodecenoic acid	0.1295
199.0847	L-Anticapsin	3.339
200.0316	4-Maleylacetoacetic acid - 2 tags / 4-Fumarylacetoacetic acid	0.62325
200.0328	2-Hydroxy-5-carboxymethylmuconate semialdehyde	0.3265
200.1387	(S)-9-Hydroxy-10-undecenoic acid	0.6675
200.1771	Dodecanoic acid	0.526
201.1359	Capryloylglycine	1.573
202.047	cis-(Homo)2-aconitic acid	0.42575
202.0952	Serylproline	0.4355
202.1199	Isomer 1 of Sebacic Acid	0.9915
202.1201	Sebacic Acid	0.3795
202.1312	Alanyl-Leucine	0.23125
202.1317	Isoleucyl-Alanine	0.41775
202.1317	Leucyl-Alanine	0.39775
202.1427	Symmetric dimethylarginine	0.16825
204.0882	L-Tryptophan / D-Tryptophan	0.3045
204.1108	N2-Acetyl-L-Hydroxylysine	0.1469
205.0572	N-Acetyl-L-erythro-4-Hydroxyglutamic acid	0.311375
206.0424	2-Methylcitric acid	0.24975
206.0578	Eugenitol	4.1305
206.0588	2-Hydroxy-3-methylbenzalpyruvic acid	2.542125
206.0945	3-Dimethylallyl-4-hydroxybenzoic acid	3.59925
207.0896	3-Phenylpropionylglycine	2.51925
207.0899	N-Acetyl-Phenylalanine	2.07025

208.0723	5-(3',4'-Dihydroxyphenyl)-gamma-valerolactone	2.0005
208.0733	Benzylsuccinic acid	2.804
208.0733	Isomer 1 of Benzylsuccinic acid	0.30725
208.0738	Sinapoyl aldehyde	1.11325
210.0523	5-Hydroxyferulic acid methyl ester	1.70225
210.0526	Vanilpyruvic acid	2.328
210.0543	Isomer 1 of 5-Hydroxyferulic acid methyl ester	1.46775
210.0847	3,4-Dihydroxyphenylvaleric acid	0.80025
210.089	3-(4-Hydroxy-3-methoxyphenyl)-2-methylpropionic acid	2.49575
210.125	(-)-Jasmonic acid / (+)-7-Isojasmonic acid	0.4705
211.0473	Betalamic acid	0.30375
211.0839	N-Acetyl-L-Noradrenaline	0.229
212.0321	5-Carboxyvanillic acid	1.108
212.0684	Vanillactic acid	0.562
212.1047	2-Amino-3,4-dimethylimidazo[4,5-f]quinoline	2.4055
212.1407	Traumatin	0.3035
212.1413	12-Oxo-9(Z)-dodecenoic acid	0.409
212.1416	Isomer 1 of Traumatin	0.48875
214.0479	2-Hydroxy-6-oxonona-2,4-diene-1,9-dioic acid	0.78525
214.1302	Prolyl-Valine	1.04975
214.1308	Isomer 1 of Prolyl-Valine	0.58725
215.0551	sn-Glycero-3-phosphoethanolamine	0.105625
216.0633	cis-(Homo)3-aconitic acid	2.373
216.064	Isomer 1 of cis-(Homo)3-aconitic acid	0.9115
216.136	Undecanedioic Acid	0.422
216.1478	Valyl-Valine	0.242
216.1717	3-Hydroxydodecanoic acid	0.99925
217.0586	2-(Hydroxymethyl)-3-(acetamidomethylene)succinic acid	1.71875
218.0907	Alanyl-Glutamic acid	0.23025

218.1149	2-Hydroxydecanedioic acid	0.3045
218.115	Isomer 1 of 2-Hydroxydecanedioic acid	1.11825
219.0847	Serylasparagine	0.667
220.0581	(-)-threo-Iso(homo)2-citric acid	1.23825
220.085	Alanyl-Methionine	0.516
222.0522	2-Succinylbenzoic acid	0.3645
222.0733	Ethyl glucuronide	1.0885
222.1005	Glycyl-Phenylalanine	0.2531
222.1048	N2'-Acetyl-5'-Hydroxykynurenamine	1.59075
225.0624	4-Amino-4-deoxychorismic acid	3.48725
226.0474	Prephenic acid	0.3635
226.0589	Uridine	0.2195
226.0827	3-(4-Hydroxy-3-methoxyphenyl)-2-methyllactic acid	1.38775
226.0951	Porphobilinogen	1.532
227.0396	N-Acetyl-2-Amino-3-carboxymuconate semialdehyde	0.4595
228.0492	L-Serine-phosphoethanolamine	0.0845
228.1358	Traumatic Acid	0.34925
228.1467	Prolyl-Leucine	0.62525
228.1468	Isoleucyl-Proline	0.65325
228.1469	Leucyl-Proline	0.64775
228.1474	Prolyl-Isoleucine	0.4655
229.1069	Prolyl-Asparagine	0.20025
229.1792	N1,N8-Diacetylspermidine	0.4005
230.0907	Prolyl-Aspartate	0.45025
230.1517	Dodecanedioic Acid	1.208
230.1625	Valyl-Leucine	0.37825
231.11	Suberylglycine	0.36025
231.1582	Gamma-Aminobutyryl-lysine	0.3605
232.106	4-(L-gamma-Glutamylamino)butanoic acid	0.32925

232.1069	Valyl-Aspartate	0.24825
232.1179	N-Acetyl-N(omega)-Hydroxyarginine	0.39825
232.1422	Leucyl-Threonine	0.32925
232.1423	Threoninyl-Leucine	0.557
232.1424	Isoleucyl-Threonine	0.29625
233.0692	2-Hydroxy-6-oxo-(2'-aminophenyl)-hexa-2,4-dienoic acid	0.249
234.0737	(R)-(Homo)3-citric acid	4.10925
234.0863	2-[(2-Aminoethylcarbamoyl)methyl]-2-hydroxybutanedioic acid	0.85525
234.1007	5-Methoxytryptophan	0.49175
236.081	L-Formylkynurenine	1.36325
236.1162	Alanyl-Phenylalanine	0.2775
237.064	N-Acetyl-2-Carboxy-2,3-dihydro-5,6-dihydroxyindole	4.399
237.0661	Isomer 1 of N-Acetyl-2-Carboxy-2,3-dihydro-5,6-dihydroxyindole	1.27925
238.0476	L-4-Chlorotryptophan	2.22725
238.0841	3,4,5-Trimethoxycinnamic Acid	1.22125
239.08	N-Acetyl-3,4-Dihydroxy-L-phenylalanine	1.38675
240.0632	(1R,6R)-6-Hydroxy-2-succinylcyclohexa-2,4-diene-1-carboxylic acid	0.3455
243.0892	gamma-Glutamyl-beta-cyanoalanine	1.09475
243.1224	Prolyl-Glutamine	0.657
243.1577	Lysyl-Proline	0.138125
244.1062	Glutamyl-Proline	0.508
244.1065	Prolyl-Glutamate	0.58425
244.1784	Isoleucyl-Isoleucine	0.441
244.1788	Leucyl-Isoleucine	0.44525
246.1002	N-acetyltryptophan	1.26075
246.1205	Isoleucyl-Aspartate	0.3915
246.1214	Leucyl-Aspartate	0.3905
246.1449	3-Hydroxydodecanedioic acid	0.53175
247.0806	Asparaginyl-Aspartic acid	0.24035

248.0641	L-beta-aspartyl-L-aspartic acid	0.057
250.0478	2-Hydroxy-6-oxo-6-(2-hydroxyphenoxy)-hexa-2,4-dienoic acid	0.56
251.079	Isomer 1 of N-Phenylacetylaspartic acid	0.55775
251.0791	Isomer 2 of N-Phenylacetylaspartic acid	0.67875
251.0796	N-Phenylacetylaspartic acid	0.12275
254.2235	(9Z)-Hexadecenoic acid	0.161
258.0599	Isogentisin	0.2385
258.0849	L-gamma-Glutamyl-(3R)-L-beta-ethynylserine	0.50725
258.1214	Saccharopine	0.383
259.153	Leucyl-Glutamine	0.42275
260.0822	1,2,3,4-Tetrahydro-b-carboline-1,3-dicarboxylic acid	3.2715
260.137	Leucyl-Glutamate	0.23425
260.1372	Isoleucyl-Glutamate	0.294525
261.0958	Glutamyl-Asparagine	0.30625
262.0144	Homovanillic acid sulfate	0.6485
262.015	3-(3,5-dihydroxyphenyl)-1-propanoic acid sulphate	0.46975
262.0952	L-cis-Cyclo(aspartylphenylalanyl)	1.0005
262.1164	Nopalinic acid	0.20725
264.1472	Phenylalanyl-Valine	0.3865
264.1472	Valyl-Phenylalanine	0.3865
266.0955	N2-Acetyl-5'-Hydroxykynurenine	1.274475
268.0582	L-Homocystine	1.10375
268.0794	3-Deoxy-D-glycero-D-galacto-2-nonulosonic acid	0.27675
268.1012	Tyrosyl-Serine	0.476
270.0869	Pinostrobin	0.5
270.2183	16-Oxopalmitic acid	0.159
272.0699	Garbanzol	0.15725
272.0893	Arbutin	1.3375
273.0998	L-Thyronine	2.06375

275.1115	Glutamyl-Glutamine	0.28925
276.0958	Gamma-Glutamylglutamic acid	0.14675
276.1321	Isomer 1 of Saccharopine	0.32375
277.0266	3,4-Dihydroxy-L-phenylalanine 3-O-sulfate	0.32
277.1059	Glutaminylmethionine	1.10275
277.1417	Methionyl-Lysine	0.30825
278.0906	Glutamylmethionine	0.91275
278.0997	Isomer 1 of Isovalerylglucuronide	0.72275
278.0999	Isovalerylglucuronide	0.81625
278.1541	alpha-CEHC	0.33575
278.1631	Leucyl-phenylalanine	0.49875
278.223	Alpha-Linolenic Acid	0.007
278.2236	Crepenynic acid	0.034
280.2382	Linoleic Acid	0.00625
281.1125	N6-Methyladenosine	1.12675
282.2541	Oleic Acid / Vaccenic Acid	0.01025
283.0898	Guanosine	0.2775
283.0921	8-Hydroxy-deoxyguanosine	0.28025
284.0751	N2-Acetyl-5-Phosphooxy-L-lysine	0.3845
288.23	10,16-Dihydroxyhexadecanoic acid	0.3755
290.0791	Fisetinidol-4beta-ol	0.36725
290.0802	Isomer 1 of Fisetinidol-4beta-ol	0.86575
290.0904	N-gamma-Glutamyl-S-allylcysteine	0.72875
291.1212	Seryl-Tryptophan	1.6655
292.1057	Canavaninosuccinic acid	1.36825
294.1228	Phenylalanyl-Glutamate	0.426
294.2182	2(R)-HOT	0.06875
294.22	9(S)-HOT	0.274
296.0882	Dehypoxanthine futalosine	1.1465

296.2342	18-Oxooleic acid	0.006725
297.1065	1-Methylguanosine	0.59425
300.0848	Dimethyl fukiic acid	0.71675
300.1207	Salidroside	1.41575
302.0999	Histidinyl-Methionine sulfoxide	1.189
303.158	gamma-Glutamylarginine	0.78775
310.1123	Tyrosyl-Glutamic acid	0.79375
312.2284	Octadec-9-ene-1,18-dioic-acid	0.28575
312.2298	(7S,8S)-DiHODE	0.5555
312.2301	5S,8R-DiHODE	0.344
314.1587	Dapdiamide B	0.60525
314.2451	9,10-DHOME	0.5215
316.2007	all-trans-18-Hydroxyretinoic acid	0.18025
316.2009	Pisiferic acid	0.532
316.2043	all-trans-4-Hydroxyretinoic acid	4.33625
316.2604	9,10-Dihydroxystearic acid	0.39525
318.2161	15-OxoETE	0.152
318.2186	Isomer 1 of 15-OxoETE	3.96125
320.0894	4-Coumaroylshikimic acid	0.8555
320.199	5'-Carboxy-alpha-chromanol	1.183
322.1309	Avenic acid A	0.521
326.0994	cis-beta-D-Glucosyl-2-hydroxycinnamic acid	0.769
328.106	Tyrosyl-Methionine sulfoxide	0.34875
330.24	9,10-Dihydroxy-12,13-epoxyoctadecanoic acid	0.4355
330.2409	9,10,13-TriHOME	0.16525
332.197	7'-Carboxy-gamma-tocotrienol	0.36225
332.2559	9,10,18-Trihydroxystearic acid	0.1025
336.0848	5-O-Caffeoylshikimic acid	4.08375
336.0848	Isomer 3 of 5-O-Caffeoylshikimic acid	1.3475

336.085	Isomer 2 of 4-Caffeoyl-1,5-quinolactone	0.56925
336.0852	Isomer 2 of 5-O-Caffeoylshikimic acid	1.113
336.0858	4-Caffeoyl-1,5-quinolactone	0.25025
336.0858	Isomer 1 of 4-Caffeoyl-1,5-quinolactone	1.87325
336.0875	Isomer 1 of 5-O-Caffeoylshikimic acid	0.9075
337.0879	S-(Hydroxymethyl)glutathione	0.35425
338.0997	p-Coumaroyl quinic acid	1.093
341.1313	6-(alpha-D-Glucosaminyl)-1D-myo-inositol	0.0445
343.1056	Caffeoyl tyrosine	0.49475
344.1083	Domesticoside	1.489
346.0876	3,4-Dihydroxyphenylethyleneglycol 4-O-glucuronide	0.11405
350.1004	3-Feruloyl-1,5-quinolactone	1.698
350.1016	4-Feruloyl-1,5-quinolactone	1.0125
354.0937	Isomer 1 of Cryptochlorogenic acid	2.57675
354.0951	Cryptochlorogenic acid	0.89775
354.0951	Chlorogenic acid	3.1465
354.0955	Isomer 2 of Chlorogenic acid	0.68125
354.0956	Isochlorogenic acid	1.17925
354.0958	Isomer 1 of 1-O-Caffeoylquinic acid	0.45375
354.0969	1-O-Caffeoylquinic acid	0.72225
360.0847	Rosmarinic acid	0.561
366.1224	Javamide-II (N-Caffeoyltryptophan)	0.136
366.1432	Tetrahydropentoxyline	0.129
368.1104	5-O-Feruloylquinic acid	0.733
368.1105	3-O-Feruloylquinic acid	0.4995
368.1106	Isomer 1 of 3-O-Feruloylquinic acid	0.592
368.1219	2-Hydroxyestradiol 17-O-sulfate	0.40275
384.1054	5-(3',4'-Dihydroxyphenyl)-gamma-valerolactone-4'-O-glucuronide	0.1635
384.126	S-Adenosyl-L-homocysteine	0.36025

398.1203	Taraxacoside	0.8115
398.1209	5-(3',4'-dihydroxyphenyl)-gamma-valerolactone-3'-O-glucuronide	0.65425
402.1165	4-Hydroxy-5-(3',5'-dihydroxyphenyl)-valeric acid-O-glucuronide	0.29075
434.1222	Naringenin 7-O-beta-D-glucoside	4.88325
